# Protective Effects of the Wenfei Buqi Tongluo Formula on the Inflammation in Idiopathic Pulmonary Fibrosis through Inhibiting the TLR4/MyD88/NF-*κ*B Pathway

**DOI:** 10.1155/2022/8752325

**Published:** 2022-02-07

**Authors:** Siyu Song, Jing Wang, Guanwen Liu, Lu Ding, Yaxin Li, Hongyu Qi, Lai Wei, Jiachao Zhao, Tian Chen, Meiru Zhao, Ziyuan Wang, Yingying Yang, Daqing Zhao, Xiangyan Li, Zeyu Wang

**Affiliations:** ^1^College of Integrated Traditional Chinese and Western Medicine, Changchun University of Chinese Medicine, Changchun, China; ^2^Department of Respiration, Affiliated Hospital of Changchun University of Chinese Medicine, Changchun, China; ^3^GCP, Affiliated Hospital of Changchun University of Chinese Medicine, Changchun, China; ^4^Jilin Ginseng Academy, Key Laboratory of Active Substances and Biological Mechanisms of Ginseng Efficacy, Ministry of Education, Jilin Provincial Key Laboratory of Bio-Macromolecules of Chinese Medicine, Changchun University of Chinese Medicine, Changchun, China; ^5^College of Traditional Chinese Medicine, Changchun University of Chinese Medicine, Changchun, China; ^6^Graduate College, Beijing University of Chinese Medicine, Beijing, China; ^7^Department of Scientific Research, Changchun University of Chinese Medicine, Changchun, China

## Abstract

**Background:**

Idiopathic pulmonary fibrosis (IPF) is a progressive disease with high mortality and poor prognosis. The prognostic signatures related to conventional therapy response remain limited. The Wenfei Buqi Tongluo (WBT) formula, a traditional Chinese medicine (TCM) formula, has been widely utilized to treat respiratory diseases in China, which is particularly effective in promoting inflammatory absorption. In this study, we aim to explore the mechanism of the WBT formula in the inhibition of inflammatory response during IPF, based on network pharmacology and *in vivo* experiments.

**Methods:**

Network pharmacology was applied to predict the changes of biological processes and potential pathways for the WBT formula against IPF. Histopathological changes, inflammatory factors (IL-6, IL-1*β*, and TNF-*α*), and the proteins of the TLR4/MyD88/NF-*κ*B pathway in bleomycin- (BLM-) induced mice model were examined by hematoxylin-eosin (H&E), Masson or immunohistochemistry staining, Western blot, and enzyme-linked immunosorbent assay analysis.

**Results:**

A total of 163 possible components and 167 potential targets between the WBT formula and IPF were obtained. The enrichments of network pharmacology showed that inflammation response, TNF, and NF-*κ*B pathways were involved in the treatment of WBT against IPF. The *in vivo* experiments indicated that the WBT formula could ameliorate inflammatory exudation and collagen deposition at a histopathology level in the BLM-induced mice model. The levels of IL-6, IL-1*β*, and TNF-*α* were reduced after the WBT formula treatment. Moreover, the expressions of phosphorylated-NF-*κ*B p65, TLR4, and MyD88 were significantly downregulated by the WBT formula, compared with the BLM-induced group.

**Conclusion:**

These results indicated that the WBT formula can suppress BLM-induced IPF in a mouse model by inhibiting the inflammation via the TLR4/MyD88/NF-*κ*B pathway. This study provides a new insight into the molecular mechanisms of the WBT formula in the application at the clinic.

## 1. Introduction

Idiopathic pulmonary fibrosis (IPF) is a chronic, progressive disease with a median survival time of three to five years since diagnosis [1]. IPF, the most common interstitial lung disease, is characterized by interstitial inflammation, fibrocyte proliferation of the alveolar wall, and fibrosis [2, 3]. Inflammatory and oxidative injuries, shortened telomeres, epithelial-mesenchymal transition (EMT), and endoplasmic reticulum stress lead to increased secretion of fibrotic factors [4]. Repeated inflammation and lung tissue injury appear to be an early-stage phenotype of pulmonary fibrosis (PF). The aberrant repair of inflammatory cells subsequently leads to the cross talk among epithelial cells, the extracellular matrix, and nearby mesenchymal cells [1]. Chronic inflammation triggered the secretion of transforming growth factor-*β* (TGF-*β*) in the alveolar compartments, which was the primary mechanism driving fibroblast activation and proliferation [5]. Inflammatory response, wounding, and oxidative stress play important roles in the prior phase of IPF [6]. It has been proven that mice deficient for the toll-like receptor 4 (TLR4) developed worse fibrosis phenotype and downregulation of cell surface hyaluronan, which has been evidenced in patients with IPF [7]. TLR4 activates the canonical NF-*κ*B pathway through MyD88 as an innate immune response, which is related to IPF [8, 9]. Importantly, the NF-*κ*B pathway as a key mediator for inflammatory response modulates the secretion of numerous cytokines and plays a key role in the inflammatory stage of PF [10, 11]. Therefore, the suppression of inflammation through the TLR4/MyD88/NF-*κ*B pathway in an early stage of the fibrosis process can effectively improve lung fibrosis.

The Wenfei Buqi Tongluo (WBT) formula is an effective prescription for treating IPF, based on a traditional and classical Chinese medicine formula, Buyang Huanwu decoction, including *Astragalus membranaceus* (Fisch.) Bunge (Huang qi), *Angelica sinensis* (Oliv.) Diels (Dang gui), *Prunus persica* (L.) Batsch (Tao ren), *Pheretima aspergillum* (E. Perrier) (Di long), *Ligusticum striatum* DC. (Chuan xiong), *Carthamus tinctorius* L. (Hong hua), and *Radix Paeoniae Rubra* (Chi shao), which was created by Qing-Ren Wang for hundreds of years during the Qing Dynasty. Buyang Huanwu decoction ameliorates PF through inhibiting the PI3K/Akt signaling pathway [12, 13]. The WBT formula is composed of 13 Chinese medicines, containing the five medicines in Buyang Huanwu decoction and other eight herbs, such as *Scutellaria baicalensis* Georgi (Huang qin), *Salvia miltiorrhiza* Bunge (Dan shen), *Polygonum cuspidatum* Siebold & Zucc (Hu zhang), *Aster tataricus* L.f. (Zi wan), *Tussilago farfara* L. (Kuan donghua), *Pinellia ternata* (Thunb.) Makino (Ban xia), *Clematis chinensis* Osbeck (Wei lingxian), and *Siegesbeckia orientalis* L. (Xi xiancao) (Table 1). As we previously reported, the WBT formula inhibited cell proliferation, morphology, and EMT in the TGF-*β*1-induced A549 cell model [14]. Multiple components in the WBT formula have been proven that they are effective in the prevention and treatment of main pathological progresses of IPF. Isorhamnetin and kaempferol are bioactive compounds from *Tussilago farfara* L. and *Pinellia ternata* (Thunb.) Makino, which reduces inflammatory cytokines and oxidative stress to protect acute lung injury in mice model by regulating the NF-*κ*B pathway [15–17]. A bioactive compound from *Ligusticum striatum* DC., ligustilide, can suppress oxidative stress and inflammation to avoid BLM-induced PF in rat model through the inhibition of the TLR4/MyD88/NF-*κ*B pathway [18]. However, the protective effects and possible mechanisms of the WBT formula in the early phase of IPF, especially the inflammatory process, have not been thoroughly explored. In this study, we first screened out IPF-related targets and potential chemical components of the WBT formula by network pharmacology, according to several common databases. Based on the prediction data, the pathway enrichment analysis was performed to elucidate the possible mechanisms of the WBT formula against IPF. Furthermore, the mice model after BLM induction for 7 days was employed to validate the anti-inflammatory effects of the WBT formula and investigate its regulatory roles on the TLR4/MyD88/NF-*κ*B pathway. Our study might provide new insights into the molecular mechanism of the WBT formula for inhibiting inflammatory response during IPF.

## 2. Materials and Methods

### 2.1. Reagents

BLM and pirfenidone (PFD) were purchased from MedChemExpress (Monmouth Junction, NJ, USA). Antibodies against TLR4 (95/120 kDa, sc-293072), MyD88 (33 kDa, sc-74532), p-NF-*κ*B p65 (65 kDa, sc-166748), and fibronectin (FN, 220 kDa, sc-8422) were purchased from Santa Cruz Biotechnology (Rosemont, IL, USA). I*κ*B*α* (39 kDa, #4814) and NF-*κ*B p65 (65 kDa, #8242) were obtained from Cell Signaling Technology (Beverly, MA, USA). Tubulin (50 kDa, 11224-1-AP) was purchased from ProteinTech (Rosemont, IL, USA).

### 2.2. Network Pharmacology Analysis for the WBT Formula and IPF

The potential gene targets of the 13 Chinese medicines in the WBT formula were identified via network pharmacology analysis. The monomer chemicals in the WBT formula were retrieved from Traditional Chinese Medicine databases (TCMSP, http://tcmspw.com/tcmsp.php) [19] and Traditional Chinese Medicine Information Database (TCMID, http://www.megabionet.org/tcmid/) [20]. In the TCMSP database, oral bioavailability ≥30% and drug-likeness ≥0.18 were conditioned to the absorption parameters for the compound screening. To collect the potential gene targets of these components of the WBT formula, the following two databases, TCMSP and SwissTargetPrediction Database (STPD, http://old.swisstargetprediction.ch/), were used [21]. Then, the Universal Protein Resource Knowledgebase (UniProt Knowledgebase, http://www.uniprot.org/, entering at Jan 2021) was applied to unify the official gene symbol, which was a collection from the candidates above. The IPF-associated target genes were acquired from GeneCards (https://www.genecards.org/) [22], the screening parameter was “relevance score ≥ 4.56.” Protein-protein interaction (PPI) data were obtained from STRING (https://cn.string-db.org/) with parameter conditions filtered by “*Homo sapiens*” (confidence score > 0.9) and visualized using Cytoscape. Finally, Gene Ontology (GO) and Kyoto Encyclopedia of Genes and Genomes (KEGG) pathways were enriched by using the Metascape (http://metascape.org/) tool.

### 2.3. Preparation of the WBT Formula

The 13 Chinese medicines present in the WBT formula were purchased from the Hongjian Pharmacy (Changchun, China), which were deposited in the Jilin Ginseng Academy, Changchun University of Chinese Medicine (Changchun, China). Raw materials of the WBT formula were codecocted in the distilled water for 1 h twice using 10 times of the total weight of the medicine mixture to obtain the aqueous extract. After filtering and centrifuging at 3,500 rpm for 15 min, the supernatants of WBT were subjected to vacuum to obtain the powder for further experiments.

### 2.4. Establishment of BLM-Induced Pulmonary Inflammation and Fibrosis and Drug Treatment

All mice (the Vital River Laboratory Animal Technology Co. Ltd, Beijing, China) were housed under standard conditions at the Experimental Animal Center, Changchun University of Chinese Medicine (Changchun, China). All experiments were approved by the Experimental Animal Administration Committee of Changchun University of Chinese Medicine (Approval No. 2021230). After three days of acclimatization, a total of 60 male C57BL/6N mice with body weight about 20 ± 2 g, aged 6–8 weeks, were randomly divided into six groups (*n* = 10): sham, BLM (3 mg/kg), WBT (3, 6, and 12 g/kg), and PFD (200 mg/kg) groups. The pulmonary inflammation and fibrosis model was established by intratracheal injection with BLM (3 mg/kg) under anesthesia by 0.3% pentobarbital sodium on day 0. The sham group was instilled with an equal volume of normal saline only. After BLM administration, mice were intragastrically administered with different doses of the WBT formula or PFD once a day for 7 consecutive days. On day 8, all the mice were sacrificed to collect serum, bronchoalveolar lavage fluid (BALF), and lung tissues for further analysis.

### 2.5. Interleukin-6 (IL-6), TNF-*α*, and Interleukin-1*β* (IL-1*β*) Measurement

According to the instructions, the IL-6 level in the serum and TNF-*α* and IL-1*β* in BALF from the mice of different groups were determined by the enzyme-linked immunosorbent assay kit (Sinobest Bio, Shanghai, China).

### 2.6. Histopathological Analysis

The mouse lung tissue was fixed using 4% formaldehyde, was embedded in paraffin, and then was cut into the slices at 4 *μ*m thickness. The lung sections were deparaffinized with xylene after being rehydrated in water by graded concentrations of ethanol solution. The sections were stained to estimate lung inflammation and fibrotic changes using H&E and Masson's trichrome stainings. For immunohistochemical (IHC) staining, the slides were submerged with a citric acid solution (pH 6.0, 0.01 mol/L; Servicebio, Wuhan, China) for 10 min for antigen retrieval. After incubation with 3% hydrogen peroxide for 25 min, the slides were incubated with primary antibodies against TLR4, MyD88, and p-NF-*κ*B overnight at 4°C and secondary antibody for 1 h. The positive staining was determined with a 3,3′-diaminobenzidine substrate and counterstained with hematoxylin. Histological images were acquired using an M8 microscope (PreciPoint, Thüringen, Germany).

### 2.7. Western Blot Analysis

Proteins from mouse lung tissues were extracted, separated on 12% SDS-PAGE gels, and transferred to the PVDF membranes (Roche, Basel, Switzerland). After blocking with 5% BSA or nonfat milk for 1.5 h, the membranes were incubated with primary antibodies against TLR4, MyD88, I*κ*B*α*, NF-*κ*B p65, p-NF-*κ*B p65, and Tubulin overnight at 4°C. After the incubation with HRP-conjugated secondary antibody (ProteinTech, IL, USA) for 1 h at room temperature, the membranes were washed three times with Tris-buffered saline with 0.1% Tween 20. The protein brands were visualized and detected using the ECL Moon kit (Beyotime Biotechnology, Shanghai, China) by a chemiluminescence detection system (ChemiDoc XRS, Bio-Rad, CA, USA). The quantification of the bands was performed using the ImageJ software (National Institutes of Health, Bethesda, MD, USA).

### 2.8. Statistical Analysis

Data were analyzed on the Prism software version 8 (GraphPad, San Diego, CA, USA). All values are expressed as the mean ± SEM. The significant differences among the groups were evaluated by one-way ANOVA with Tukey's post hoc test. The results are accepted as the level of significance was set at *P* value of < 0.05.

## 3. Results

### 3.1. The Activated Compounds and Potential Targets of the WBT Formula against IPF Are Predicted by Network Pharmacology

**Figure 1:**
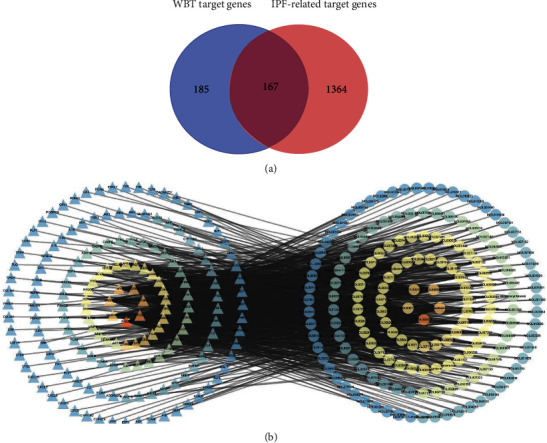
Screening of potential targets and the compound-target-disease network. (a) Venn chart of potential target genes between the WBT formula and IPF. A total of 352 genes of the components from the WBT formula and 1,531 disease genes of IPF were predicted and contributed to 167 shared targets, as potential targets of the WBT formula treating IPF. (b) Bioactive components of the WBT formula-potential targets network. The left triangles represent treatment targets of the WBT formula for IPF, the right circles represent the bioavailable components of the WBT formula.

Using the TCMSP and TCMID databases, 243 candidate compounds were found in the WBT formula. Among these components, 20, 36, 65, 10, 2, 7, 23, 10, 19, 22, 13, 7, and 9 compounds were identified in *Astragalus membranaceus* (Fisch.) Bunge, *Scutellaria baicalensis* Georgi, *Bunge Salvia miltiorrhiza* Bunge, *Polygonum cuspidatum* Siebold & Zucc, *Angelica sinensis* (Oliv.) Diels, *Ligusticum striatum* DC., *Prunus persica* (L.) Batsch, *Pheretima aspergillum* (E. Perrier), *Aster tataricus* L.f., *Tussilago farfara* L., *Pinellia ternata* (Thunb.) Makino, *Clematis chinensis* Osbeck, and *Siegesbeckia orientalis* L., respectively (Table 2). Among these compounds, 40 compounds with the repeated names and 38 compounds without targets were deleted to obtain 165 compounds for further analysis. In the WBT formula, 352 targets of these 165 components (Supplementary Table 1) were predicted at TCMID and STPD websites. Then, we input 352 targets into the UniProt Knowledgebase website to unify the standard nomenclature. Meanwhile, 1,531 pathogenic genes-related with IPF were collected from the databases mentioned above (Supplementary Table 2). After the overlapping analysis, 167 intersection targets, as the potential targets of the WBT formula for treating IPF, were acquired and shown in a Venn diagram (Figure 1(a)). Furthermore, two compounds not for 167 targets of IPF were screened out to obtain 163 candidate compounds. In addition, those intersection targets were used to build the network diagram of the components of the WBT formula and their targets, which were composed of 330 nodes and 999 edges (Figure 1(b) and Supplementary Table 3). Collectively, 163 candidate compounds and 167 potential targets of the WBT formula against IPF were predicted by a network pharmacology-based method.

### 3.2. Potential Pathways and Gene Functional Enrichments for the WBT Formula against IPF

**Figure 2:**
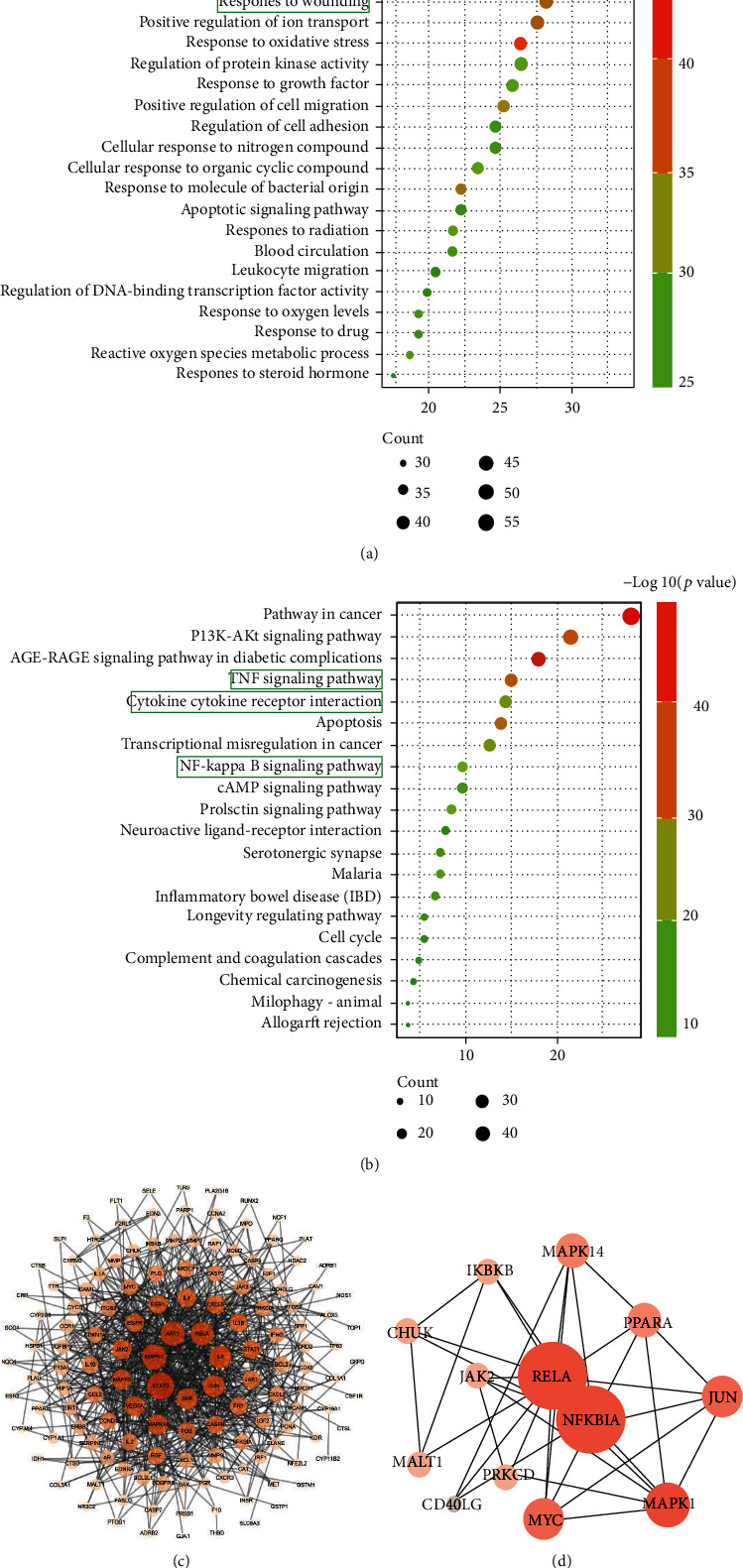
The GO and KEGG pathway enrichment analysis of the key targets for the WBT formula. GO enrichment analysis showing the top 20 biological processes (a) and KEGG pathway analysis (b) for 167 shared targets from the WBT formula and IPF-related target genes. (c) The PPI network of targets for the WBT formula against IPF was constructed by the Cytoscape software. (d) The PPI network of an NF-*κ*B pathway-related gene from (c) is shown. The size and color depth of nodes are positively correlated with their degrees, respectively.

The GO annotation and KEGG pathway enrichments for 167 overlapping targets showed the top 20 significantly enriched terms in the biological process (BP), and cell component (CC) and molecular function (MF) categories are shown in Figure 2(a) and Supplementary Figure 1. As suggested from the results, 167 key targets showed tight relations to the major BP, such as inflammatory response, response to wounding, response to oxidative stress, and regulation of cell adhesion. The WBT formula was reported for integrating multiple signaling pathways in the fibrosis process, inflammatory response, and cancers. The enrichment of target-pathway was built to delve into the mechanisms of potential targets acting on their corresponding signal pathways (Figure 2(b)). The PPI network for therapeutic targets of the WBT formula against IPF was constructed (Figure 2(c)). Furthermore, the therapeutic effect of the WBT formula on IPF was most likely achieved through modulating multiple pathways such as the PI3K-Akt, TNF, and NF-*κ*B pathways. Among these pathways, the NF-*κ*B pathway might be the core potential pathway of the WBT formula for inhibiting the inflammatory response, wounding response, and subsequent fibrosis. The targets of the NF-*κ*B pathway subnetwork were built and are shown in Figure 2(d). Based on the results of the network pharmacology enrichment analysis, we further examined whether the protective effect of the WBT formula against inflammation and subsequent fibrosis were dependent on the regulation of the NF-*κ*B-mediated inflammatory pathways.

### 3.3. Anti-Inflammatory and Fibrotic Effects of the WBT Formula on BLM-Induced Mice

**Figure 3:**
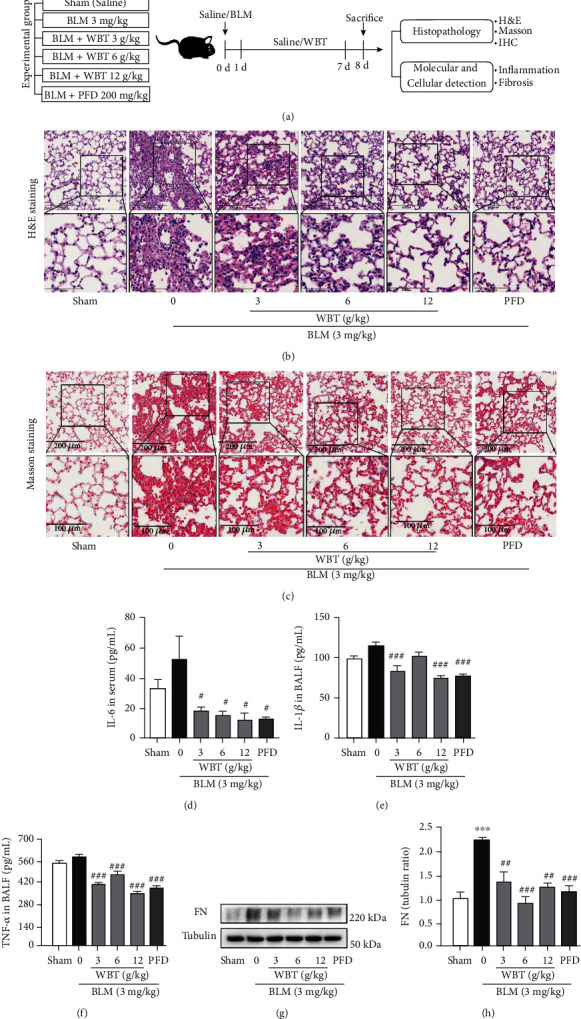
The WBT formula alleviates inflammation and fibrosis in a mouse model induced by BLM. (a) Diagram describing the protocol of animal experiment (*n* = 10 mice in each group). (b) H&E staining for observing inflammatory cell infiltration of lung tissues. (c) Masson's trichrome staining for detecting collagen fibers. (d–f) Serum IL-6 level and IL-1*β*, and TNF-*α* in BALF in different groups was measured by ELISA commercial kits. (g, h) The level of FN in lung tissues was measured by Western blot analysis. The bands from three independent experiments were semiquantitatively analyzed by using the ImageJ software, normalized to Tubulin density. Sham: sham surgery with intratracheal administration of normal saline (NS) + oral NS for 7 days; BLM (intratracheal administration of BLM (3 mg/kg) + oral NS); BLM induction and WBT/PFD treatment: BLM + WBT (3, 6, or 12 g/kg)/PFD (pirfenidone 200 mg/kg) for 7 days. Scale bar = 200 *μ*m (upper image), 100 *μ*m (lower image). ^∗∗∗^*P* < 0.001, compared to the sham group; ^#^*P* < 0.05, ^##^*P* < 0.01, and ^###^*P* < 0.001 compared with the BLM group. All data were analyzed using one-way ANOVA followed by Tukey's test.

To further validate the mechanism of the WBT formula for preventing inflammatory response during lung fibrosis, the protocol for animal group, drug administration, and mechanism experiment were designed and are visualized in Figure 3(a). As shown in Figures 3(b) and 3(c), histopathological changes with inflammatory and fibrotic responses due to BLM induction showed the gathering of multiple inflammatory cells and collagen fibrogenesis in alveolar spaces after 7 days. Alveolar infiltration in the model group was suppressed in a dose-dependent manner after treatment with the WBT formula (Figure 3(b)). Masson's trichrome staining reflected collagen accumulation, as an indicator for lung fibrosis. The newly formed collagen fibers were decreased by the treatment of the WBT formula and PFD. The WBT formula at the dose of 12 g/kg and PFD effectively inhibited the formation of collagen fiber in the alveoli (Figure 3(c)), compared to the BLM-induced group. Subsequently, we examined the content of the IL-6 level in the serum and the levels of IL-1*β* and TNF-*α* in BALF from different groups. We found that WBT or PFD treatment significantly inhibited serum IL-6 level, compared with the BLM-induced group (Figure 3(d)). Importantly, the contents of IL-1*β* and TNF-*α* in BALF induced by BLM were lower than that of WBT or PFD treatment (Figures 3(e) and 3(f)). In addition, Western blot analysis showed that the expression of FN, a key molecule involved in fibrosis, was upregulated by BLM induction, which was obviously suppressed by WBT or PFD treatment (Figures 3(g) and 3(h)). Together, these data indicated that the WBT formula can inhibit inflammatory and fibrotic responses in the BLM-induced mouse model.

### 3.4. The WBT Formula Attenuates BLM-Induced Inflammatory Response by Inhibiting the TLR4/MyD88/NF-*κ*B Pathway to Protect Lung Tissue from Fibrosis

**Figure 4:**
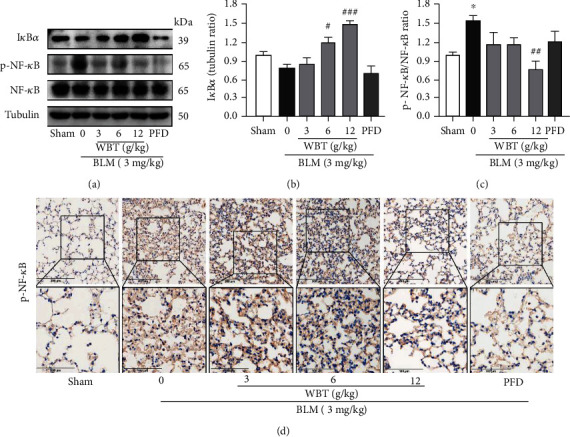
The WBT formula alleviated the NF-*κ*B signaling pathway in BLM-induced mouse model. (a) Protein levels of phosphorylated- NF-*κ*B p65 (p-NF-*κ*B p65), total p65, and I*κ*B*α* were measured with Western blotting. Tubulin was a loading control. (b, c) The bands of I*κ*B*α*, p-NF-*κ*B p65, and p65 were semiquantitatively analyzed by using the ImageJ software, normalized to Tubulin density, and calculated relative I*κ*B*α* expression and the ratio of p-NF-*κ*B p65 and p65. (d) The p-NF-*κ*B p65 expression in lung tissues from different groups was determined by IHC staining. Scale bar: 200 *μ*m (upper image), 100 *μ*m (lower image). BLM: bleomycin. Sham: sham surgery with intratracheal administration of normal saline (NS) + oral NS for 7 days; BLM (intratracheal administration of BLM (3 mg/kg) + oral NS); BLM induction and WBT/PFD treatment: BLM (3 mg/kg) + WBT (3, 6, or 12 g/kg)/PFD (pirfenidone, 200 mg/kg) for 7 days, *n* = 10. ^∗^*P* < 0.001, compared to the sham group; ^#^*P* < 0.05, ^##^*P* < 0.01, and ^###^*P* < 0.001 compared with the BLM group.

**Figure 5:**
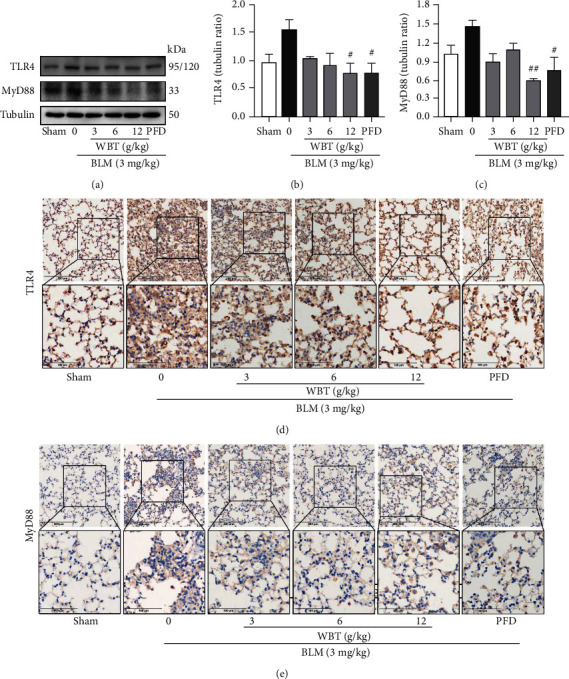
The WBT formula decreased the protein expression of the TLR4/MyD88 pathway in mouse lung tissues induced by BLM. (a) Protein levels of TLR4 and MyD88 were measured by Western blot analysis. (b, c) Quantification of relative expression of TLR4 or MyD88 was performed by densitometric analysis after the normalization of Tubulin. Tubulin was a loading control. (d, e) The levels of TLR4 and MyD88 were detected by immunohistochemical staining in lung tissues from sham (sham surgery with intratracheal administration of normal saline (NS) + oral NS), BLM (intratracheal administration of BLM (3 mg/kg) + oral NS), and WBT (intratracheal administration of BLM (3 mg/kg) + 3, 6, or 12 g/kg WBT) or PFD (intratracheal administration of BLM (3 mg/kg) + pirfenidone 200 mg/kg) for 7 days, *n* = 10. BLM: bleomycin. Scale bar: 200 *μ*m (upper image), 100 *μ*m (lower image). ^#^*P* < 0.05 and ^##^*P* < 0.01 compared with the BLM group.

To further validate the mechanism of the WBT formula for inflammatory response from the prediction, Western blot and IHC staining methods were used to analyze the NF-*κ*B inflammatory pathway for clarifying the mechanism of the WBT formula. Western blotting revealed that I*κ*B*α* was reduced by BLM, while NF-*κ*B p-p65/p65 was upregulated in the BLM-induced lung tissues (Figures 4(a)–4(c)). WBT treatment promoted the expression of I*κ*B*α* and inhibited the phosphorylation of NF-*κ*B p65, compared with the BLM group (Figures 4(a)–4(c)). Similarly, the increased phosphorylation of NF-*κ*B p65 induced by BLM in lung tissues was inhibited by the WBT formula according to IHC staining (Figure 4(d)). Recent studies have shown that TLR4 activity is crucial for inflammation and PF [23], which is upstream of the NF-*κ*B pathway. MyD88 can be recruited to mediate the release of proinflammatory cytokines when the TLR4 pathway is activated [24, 25]. Therefore, we used Western blot and IHC staining to further investigate the effect of the WBT formula on the levels of TLR4 and MyD88 in the BLM-induced lung tissues. As shown in Figures 5(a)–5(c), Western blot analysis showed that BLM increased the expressions of TLR4 and MyD88 in the lung tissues, which were significantly downregulated after WBT formula intervention. For the IHC staining shown in Figure 5(d), similar results were found that WBT treatment significantly suppressed the BLM-induced increases of TLR4 and MyD88. Taken together, the WBT formula can inhibit the TLR4/MyD88/NF-*κ*B signaling pathway to reduce inflammatory response in a BLM-induced mouse model with IPF.

## 4. Discussion

**Figure 6:**
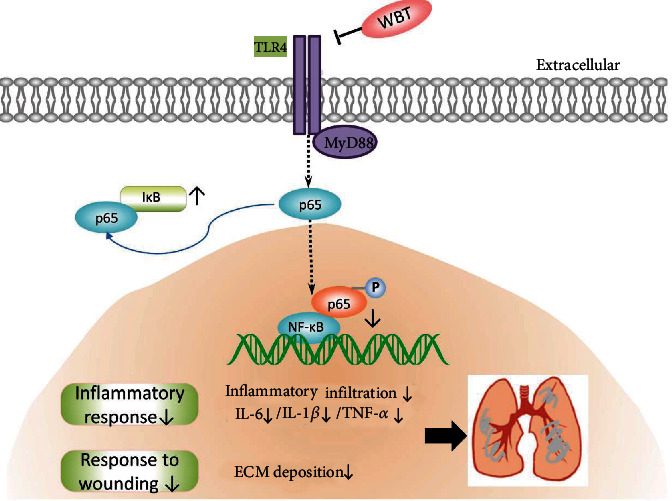
The potential mechanism of the WBT formula for inhibiting the TLR4/MyD88/NF-*κ*B pathway-mediated inflammation against IPF.

In the early stages following inflammation, fibroblasts and myofibroblasts provide a tissue scaffold for repairing the injured alveolar epithelial cell [26]. The inflammatory response is the initial pathogenesis of IPF, which stimulates the secretion of profibrotic cytokines to initiate fibroblast activation. Hence, the suppression of inflammation could slow down the IPF process [27]. The potential targets of the WBT formula for inhibiting inflammatory response were predicted using a network pharmacology approach. The WBT formula is a TCM and has extensively achieved efficacy in the Affiliated Hospital of Changchun University of Chinese Medicine (Changchun, China) for the prevention and treatment of IPF. However, its exact mechanism of anti-inflammatory action on IPF is still unclear. This study provides experimental evidence for the inhibitory effect of the WBT formula against inflammatory response by inactivating the TLR4/MyD88/NF-*κ*B pathway, which could be a potential mechanism of the WBT formula treatment for IPF patients (Figure 6).

Some herbs and bioactive components have been shown to possess pharmacologic activities for the treatment of IPF. *Astragalus membranaceus* (Fisch.) Bunge is a kind of the most widely used traditional Chinese herbal medicines for antioxidant and anti-inflammatory effects [28]. Extracts of *Scutellaria baicalensis* Georgi and its major chemical constituents have been reported to possess antioxidant and anti-inflammatory functions [29], which promoted wound healing by suppressing the secretion of inflammatory cytokines [28]. After the prediction by network pharmacology, the degrees representing the connections of the nodes with direct neighbors were calculated to estimate the significance of the nodes in a network between the compounds and the targets [30]. In the present study, some targets with higher degree values are important for the network. The top ten degree-ranked compounds are quercetin (MOL000098), xanthinin, luteolin (MOL000006), kaempferol (MOL000422), wogonin (MOL000173), tanshinone IIA (MOL007154), baicalein (MOL002714), beta-sitosterol (MOL000358), 7-O-methylisomucronulatol (MOL000378), and dihydrotanshinlactone (MOL007100), indicating their essential roles of these compounds for the target network of the inflammatory response during IPF. Certainly, those components in the WBT formula need to require LC-MS methods to identify the active compounds in further experiments.

The early stage of IPF is manifested inflammation, especially in the mouse model after BLM for 7 days. According to recent studies and our results, the TLR4/MyD88/NF-*κ*B p65 signaling pathway appears to have potential functions in IPF [18, 31]. In our study, some compounds in the WBT formula have been shown to inhibit the TLR4/MyD88/NF-*κ*B pathway. Researchers have found that quercetin has anti-inflammatory effects and can reduce the inflammatory response in a variety of diseases and inhibit inflammation-related signaling pathways [32–34]. Luteolin can inhibit TLR4 activation in multiple diseases [35–37]. To be of worth, PI3K/Akt, AGE-RAGE, and other pathways were also predicted by network pharmacology, which is important in the multiple stages of IPF progression and requires further experimental validation.

## 5. Conclusion

This research is aimed at exploring the mechanism of the WBT formula using a network pharmacology analysis in the early stage of IPF. After the prediction, 163 candidate compounds and 167 key targets of the WBT formula against IPF were identified. The inflammatory response, TNF, and NF-*κ*B pathways were considered the potential pathway of the WBT formula, which was validated using a mouse model of IPF. The results indicated that the WBT formula can suppress IPF progression by inhibiting the TLR4/MyD88/NF-*κ*B pathway-mediated inflammation. This study provides a new insight to the molecular mechanisms of the WBT formula in the application at the clinic.

## Figures and Tables

**Table 1:** The compositions of the WBT formula.

Chinese name	Latin name	Family	Weight (g)	Part used	Voucher specimen
Huang qi	*Astragalus membranaceus* (Fisch.) Bunge	Leguminosae	40	Root	201213-1
Huang qin	*Scutellaria baicalensis* Georgi	Lamiaceae	20	Root	201213-2
Dan shen	*Salvia miltiorrhiza* Bunge	Lamiaceae	20	Root	201213-3
Hu zhang	*Polygonum cuspidatum* Siebold & Zucc	Polygonaceae	15	Rhizome	201213-4
Dang gui	*Angelica sinensis* (Oliv.) Diels	Apiaceae	15	Root	201213-5
Chuan xiong	*Ligusticum striatum* DC.	Umbelliferae	15	Root	201213-6
Tao ren	*Prunus persica* (L.) Batsch	Rosaceae	10	Seed	201213-7
Di long	*Pheretima aspergillum* (E. Perrier)	Megascolecidae	10	Whole animal	201213-8
Zi wan	*Aster tataricus* L.f.	Compositae	15	Rhizome and root	201213-9
Kuan donghua	*Tussilago farfara* L.	Compositae	15	Flower bud	201213-10
Ban xia	*Pinellia ternata* (Thunb.) Makino	Araceae	9	Tuber	201213-11
Wei lingxian	*Clematis chinensis* Osbeck	Ranunculaceae	15	Root	201213-12
Xi xiancao	*Siegesbeckia orientalis* L.	Asteraceae	15	Above ground part	201213-13

**Table 2:** The possible components of the WBT formula were predicted by network pharmacology.

Chinese medicines	Number	Components
*Astragalus membranaceus* (Fisch.) Bunge	20	Mairin, jaranol, hederagenin, quercetin, isorhamnetin, 3,9-di-O-methylnissolin, 5′-hydroxyiso-muronulatol-2′,5′-di-O-glucoside, 7-O-methylisomucronulatol, 9,10-dimethoxyp-terocarpan-3-O-*β*-D-glucoside, bifendate, etc.
*Scutellaria baicalensis* Georgi	36	Diop, panicolin, skullcapflavone II, baicalein, supraene, carthamidin, norwogonin, salvigenin, ent-epicatechin, sitosterol, etc.
*Salvia miltiorrhiza* Bunge	65	Salvilenone, salviolone, sugiol, luteolin, miltipolone, miltirone, baicalin, manool, digallate, sugiol, etc.
*Polygonum cuspidatum* Siebold & Zucc	10	6,8-Dihydroxy-7-methoxyxanthoneluteolin, beta-sitosterol, physciondiglucoside, torachrysone-8-O-beta-D-(6′-oxayl)-glucoside, quercetin, rhein, (+)-catechin, picralinal, physovenine, etc.
*Angelica sinensis* (Oliv.) Diels	2	Stigmasterol, beta-sitosterol
*Ligusticum striatum* DC.	7	Mandenol, myricanone, perlolyrine, senkyunone, wallichilide, sitosterol, FA
*Prunus persica* (L.) Batsch	23	Hederagenin, beta-sitosterol, campesterol, 3-O-p-coumaroylquinic acid, sitosterol alpha1, gibberellin 7, gibberellin 17, 2,3-didehydro GA70, gibberellin A44, populoside_qt, etc.
*Pheretima aspergillum* (E. Perrier)	10	4-Guanidino-1-butanol, cholesterol, cholesteryl ferulate, guanidine, guanine(1,7-dihydro-form), guanosine, hypoxanthine, hyrcanoside, xanthine, xanthinin, etc.
*Aster tataricus* L.f.	19	Rabdosinatol, shionone, galangin, isorhamnetin, beta-sitosterol, epifriedelanol acetate, kaempferol, spinasterol, luteolin, quercetin, etc.
*Tussilago farfara* L.	22	Tussilagolactone, beta-sitosterol, taraxanthin, kaempferol, quercetin, senkirkine, tussilagin, femara, methyl 3-o-caffeoylquinate, alpha-Carotene-5,6-epoxide, etc.
*Pinellia ternata* (Thunb.) Makino	13	Cavidine, gondoic acid, coniferin, baicalein, beta-sitosterol, cycloartenol, baicalin, stigmasterol, 24-ethylcholest-4-en-3-one, 10,13-eicosadienoic, etc.
*Clematis chinensis* Osbeck	7	(4aS,6aR,6aS,6bR,8aR,10R,12aR,14bS)-10-hydroxy-2,2,6a,6b,9,9,12aheptamethyl-1,3,4,5,6,6a,7,8,8a,10,11,12,13,14b-tetradecahydropicene-4a-carboxylic acid, (6Z,10E,14E,18E)-2,6,10,15,19,23-hexamethyltetracosa-2,6,10,14,18,22-hexaene, beta-sitosterol, stigmasterol, clematosideA'_qt, embinin, heptyl phthalate
*Siegesbeckia orientalis* L.	9	Stigmasterol, hederagenin, beta-sitosterol, 15alpha-Hydroxy-ent-kaur-16-en-19-oic acid, vernolic acid, coronaridine, siegesesteric acid II, siegesmethyletheric acid, (1R)-1-[(2S,4aR,4bS,7R,8aS)-7-hydroxy-2,4b,8,8-tetramethyl-4,4a,5,6,7,8a,9,10-octahydro-3H-phenanthren-2-yl]ethane-1,2-diol

## Data Availability

The datasets used and/or investigated during the current study are available from the corresponding authors upon reasonable request.

## Supplementary Materials

Supplementary Materials Supplementary Figure 1: GO analysis for the 167 shared targets from WBT formula and IPF-related target genes. (a) Molecular function. (b) Cell component. Supplementary Table 1: the possible targets of 165 compounds from the WBT formula. Supplementary Table 2: the target genes of idiopathic pulmonary fibrosis. Supplementary Table 3: a total of 167 potential targets of 163 candidate compounds in the WBT formula were obtained by network pharmacology.
