# Supplementary material for: Protective Effects of the Wenfei Buqi Tongluo Formula on the Inflammation in Idiopathic Pulmonary Fibrosis through Inhibiting the TLR4/MyD88/NF-κB Pathway
Source: Biomed Res Int. 2022 Feb 7;2022:8752325. doi: 10.1155/2022/8752325 (PMC8843962; doi:10.1155/2022/8752325)
Supplement: Supplementary Materials — Supplementary Figure 1: GO analysis for the 167 shared targets from WBT formula and IPF-related target genes. (a) Molecular function. (b) Cell component. Supplementary Table 1: the possible targets of 165 compounds from the WBT formula. Supplementary Table 2: the target genes of idiopathic pulmonary fibrosis. Supplementary Table 3: a total of 167 potential targets of 163 candidate compounds in the WBT formula were obtained by network pharmacology. [file 8752325.f1.docx]

**Supplementary information**

**Protective effects of the Wenfei Buqi Tongluo formula on the inflammation in idiopathic pulmonary fibrosis through inhibiting the TLR4/MyD88/NF-κB pathway**

Siyu Song^1*^, Jing Wang^2*^, Guanwen Liu^3^, Lu Ding^4^, Yaxin Li^1^, Hongyu Qi^4^, Lai Wei^5^, Jiachao Zhao^1^, Tian Chen^1^, Meiru Zhao^1^, Ziyuan Wang^5^, Yingying Yang^6^, Daqing Zhao^4^, Xiangyan Li^4#^, Zeyu Wang^7#^

^1^College of Integrated Traditional Chinese and Western Medicine, Changchun University of Chinese Medicine, Changchun, China. ^2^Department of Respiration, Affiliated Hospital of Changchun University of Chinese Medicine, Changchun, China. ^3^GCP, Affiliated Hospital of Changchun University of Chinese Medicine, Changchun, China. ^4^Jilin Ginseng Academy, Key Laboratory of Active Substances and Biological Mechanisms of Ginseng Efficacy, Ministry of Education, Jilin Provincial Key Laboratory of Bio-Macromolecules of Chinese Medicine, Changchun University of Chinese Medicine, Changchun, China. ^5^College of Traditional Chinese Medicine, Changchun University of Chinese Medicine, Changchun, China. ^6^Graduate College, Beijing University of Chinese Medicine, Beijing, China. ^7^Department of Scientific Research, Changchun University of Chinese Medicine, Changchun, China.

*SS and JW contributed equally to this article;

^#^Corresponding author: Xiangyan Li, Jilin Ginseng Academy, Key Laboratory of Active Substances and Biological Mechanisms of Ginseng Efficacy, Ministry of Education, Jilin Provincial Key Laboratory of Bio-Macromolecules of Chinese Medicine, Changchun University of Chinese Medicine, No. 1035 Boshuo Road, Changchun130017, China. Email: xiangyan_li1981@163.com. Zeyu Wang, Department of Scientific Research, Changchun University of Chinese Medicine, Changchun, No. 1035 Boshuo Road, Changchun130017, China. Email: zeyu781022@163.com.


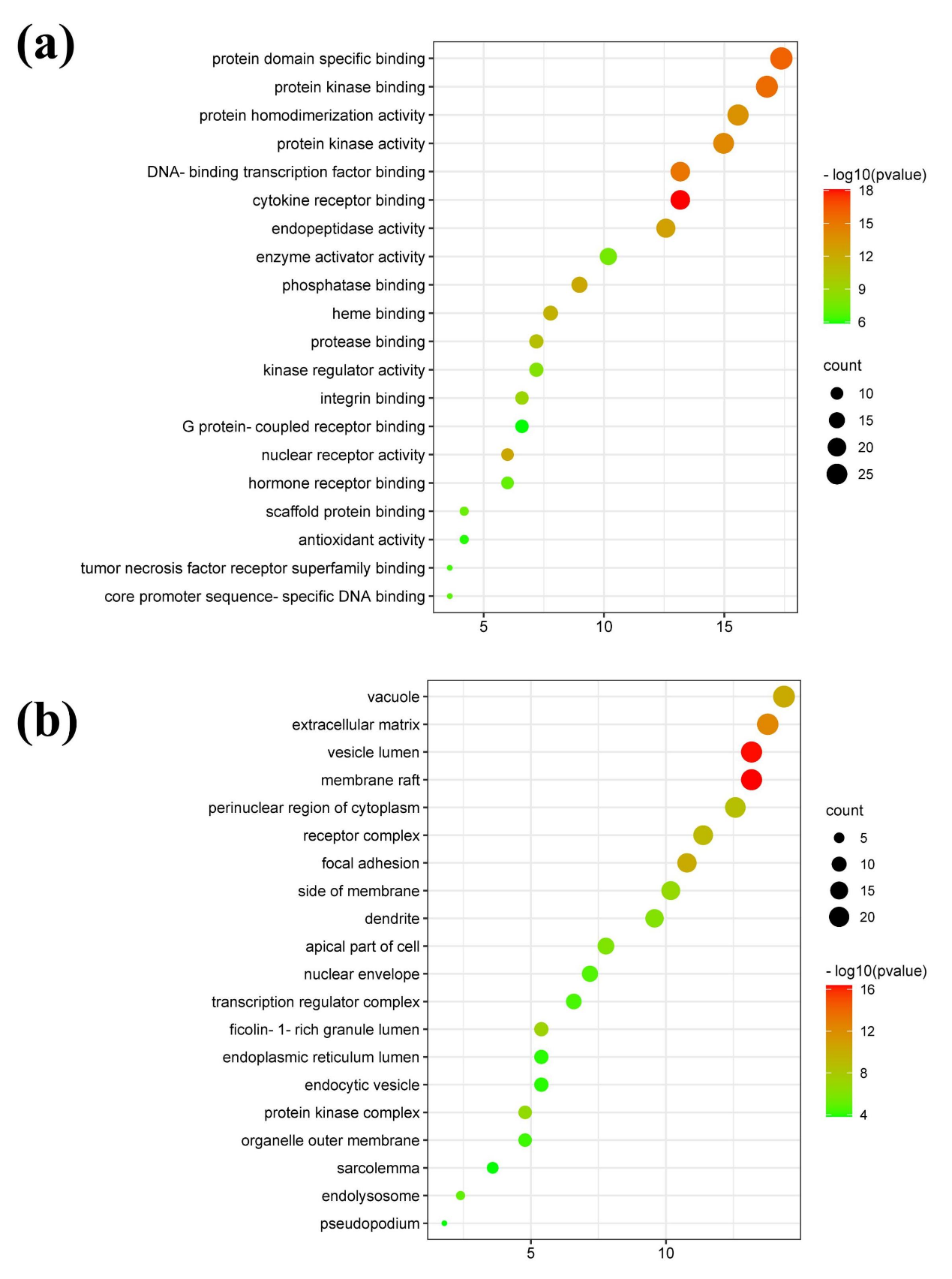


**Supplementary Figure 1**: **GO analysis for the 167 shared targets from WBT formula and IPF-related target genes.** (a) Molecular function; (b) Cell component.

**Supplementary Table 1:** The possible targets of 165 compounds from WBT formula.

| **Compounds** | **Number** | **Gene** |  |  |
| --- | --- | --- | --- | --- |
| Mairin | 1 | PGR |  |  |
| Jaranol | 8 | PTGS1, AR, SCN5A, PTGS2, ESR2, CHEK1, PRSS1, NCOA2 |  |  |
| (3S,8S,9S,10R,13R,14S,17R)-10,13-dimethyl-17-[(2R,5S)-5-propan- 2-yloctan-2-yl]-2,3,4,7,8,9,11,12,14,15,16,17-dodecahydro-1H- cyclopenta[a]phenanthren-3-ol | 1 | PGR |  |  |
| Isorhamnetin | 23 | PTGS1, ESR1, AR, PPARG, PTGS2, ESR2, MAPK14, GSK3B, PRSS1, CCNA2, NCOA2, PPARD, CHEK1, AKR1B1, NCOA1, F7, ACHE, GABRA1, MAOB, GRIA2, RELA, NCF1, OLR1 |  |  |
| 3,9-di-O-methylnissolin | 18 | PTGS1, CHRM3, CHRM1, ESR1, ADRB1, SCN5A, PTGS2, HTR3A, ADRA2C, RXRA, ACHE, ADRA1B , ADRB2, ADRA1D, OPRM1, GABRA1, PRSS1, NCOA2 |  |  |
| 7-O-methylisomucronulatol | 31 | PTGS1, CHRM3, KCNH2, CHRM1, ESR1, AR, ADRB1,  SCN5A, PPARG, CHRM5, PTGS2, ADRA2C, CHRM4,  RXRA, OPRD1, ADRA1A, CHRM2, ADRA1B, SLC6A3,  ADRB2, ADRA1D, SLC6A4, ESR2, GABRA1, MAPK14,  GSK3B, CHEK1, RXRB, PRSS1, CCNA2, NCOA2 |  |  |
| 9,10-dimethoxypterocarpan-3-O-β-D-glucoside | 2 | PTGS2, NCOA2 |  |  |
| (6aR,11aR)-9,10-dimethoxy-6a,11a-dihydro-6H- benzofurano[3,2-c]chromen-3-ol | 17 | PTGS1, CHRM3, CHRM1, ESR1, SCN5A, PTGS2, HTR3A, RXRA, ACHE, ADRA1B, ADRB2, ADRA1D, GABRA1, PRSS1, NCOA2, NCOA1, CHRM4 |  |  |
| Bifendate | 4 | PTGS2, KDR, MET, PTGS1 |  |  |
| Formononetin | 29 | PTGS1, CHRM1, ESR1, AR, PPARG, PTGS2, ADRA1A, SLC6A3, ADRB2, SLC6A4, ESR2, PTGS1, CHRM1, ESR1, AR, PPARG, PTGS2, RXRA, ADRA1A, SLC6A3, ADRB2, SLC6A4, ESR2, JUN, PPARG, IL4, ND6, HSD3B2, HSD3B1 |  |  |
| Calycosin | 14 | PTGS1, ESR1, AR, PPARG, PTGS2, RXRA, ESR2, MAPK14, GSK3B, CHEK1, PRSS1, CCNA2, NCOA2, ADRB2 |  |  |
| 1,7-Dihydroxy-3,9-dimethoxy pterocarpene | 3 | PTGS2, RXRA, PRSS1 |  |  |
| NEOBAICALEIN | 12 | KCNH2, ESR1, AR, SCN5A, PPARG, PTGS2, F7, ESR2, GSK3B, CHEK1, PRSS1, NCOA2 |  |  |
| Panicolin | 8 | PTGS1, AR, SCN5A, PTGS2, ESR2, CHEK1, PRSS1, NCOA1 |  |  |
| 5,7,4'-trihydroxy-8-methoxyflavanone | 2 | PTGS1, PTGS2 |  |  |
| Skullcapflavone II | 11 | PTGS1, KCNH2, AR, SCN5A, PTGS2, F7, KDR, CACNA2D1, PRSS1, NCOA2, NCOA1 |  |  |
| DIHYDROOROXYLIN | 7 | PTGS1, SCN5A, PTGS2, RXRA, ADRA1B, ADRB2, NCOA1 |  |  |
| (2R)-7-hydroxy-5-methoxy-2-phenylchroman-4-one | 15 | PTGS1, CHRM3, CHRM1, ESR1, SCN5A, PTGS2, RXRA, ADRA1A, ADRA1B, SLC6A3, ADRB2, SLC6A4, GABRA1, PKIA, MAOB |  |  |
| Salvigenin | 10 | PTGS1, SCN5A, PTGS2, RXRA, ACHE, ADRA1B, ADRB2, PRSS1, NCOA2, F7 |  |  |
| Ent-Epicatechin | 3 | PTGS1, ESR1, PTGS2 |  |  |
| 5,2',6'-Trihydroxy-7,8-dimethoxyflavone | 8 | PTGS1, AR, SCN5A, PTGS2, ESR2, CHEK1, PRSS1, NCOA2 |  |  |
| Moslosooflavone | 15 | PTGS1, AR, SCN5A, PPARG, PTGS2, RXRA, ESR2, GABRA1, MAPK14, GSK3B, CHEK1, PRSS1, NCOA1, ADRA1B, ADRB2 |  |  |
| Bis[(2S)-2-ethylhexyl] benzene-1,2-dicarboxylate | 1 | SCN5A |  |  |
| Diop | 3 | SCN5A, ADRB2, CHRM3 |  |  |
| Epiberberine | 7 | KCNH2, ESR1, AR, PTGS2, RXRA, PRSS1, NCOA2 |  |  |
| Oroxylin a | 17 | PTGS1, AR, SCN5A, PTGS2, RXRA, ADRA1B, ADRB2, PRSS1, NCOA1, NCOA2, PKIA, BCL2, IL6, CASP3, CYP1A2, CCNB1, CYP2C9 |  |  |
| Eriodyctiol (flavanone) | 3 | PTGS1, PTGS2, NCOA2 |  |  |
| Carthamidin | 2 | PTGS1, PTGS2 |  |  |
| Dihydrobaicalin_qt | 2 | PTGS1, PTGS2 |  |  |
| Norwogonin | 5 | PTGS1, AR, PPARG, PTGS2, CHEK1 |  |  |
| 11,13-Eicosadienoic acid, methyl ester | 1 | NCOA2 |  |  |
| Rivularin | 12 | PTGS1, KCNH2, AR, SCN5A, PTGS2, F7, KDR, RXRA, ESR2, PRSS1, NCOA2, NCOA1 |  |  |
| 5,7,2',6'-Tetrahydroxyflavone | 3 | PTGS1, AR, PTGS2 |  |  |
| 5,7,4'-trihydroxy-6-methoxyflavanone | 12 | PTGS1, PTGS2, PTGS1, ESR1, AR, PPARG, PTGS2, MAPK14, GSK3B, CHEK1, PRSS1, NCOA2 |  |  |
| Acacetin | 17 | PTGS1, AR, PTGS2, PRSS1, NCOA2, NCOA1, CHEK1, ADRB2, RELA, BCL2, CDKN1A, BAX, CASP3, TP63, CASP8, FASN, FASLG |  |  |
| 5,7,2,5-tetrahydroxy-8,6-dimethoxyflavone | 4 | AR, PTGS2, PRSS1, NCOA2 |  |  |
| 5,2'-Dihydroxy-6,7,8-trimethoxyflavone | 12 | PTGS1, KCNH2, AR, SCN5A, PTGS2, F7, ESR2, PPARD,  PRSS1, NCOA2, KDR, NCOA1 |  |  |
| Wogonin | 37 | PTGS1, ESR1, AR, SCN5A, PPARG, PTGS2, RXRA, MAPK14, GSK3B, CHEK1, PRSS1, ADRB2, GABRA1, RELA, AKT1, CCND1, BCL2, CDKN1A, EIF6, BAX, CASP9, KDR, TNFSF15, JUN, IL6, AHSA1, CASP3, TP63, BBC3, TEP1, MMP1, CCL2, PRKCD, PTGER3, FN1, CXCL8, MCL1 |  |  |
| Coptisine | 7 | PTGS1, KCNH2, ESR1, AR, SCN5A, PTGS2, PRSS1 |  |  |
| Salvilenone | 7 | PTGS1, ESR1, AR, CHRM5, PTGS2, HTR3A, ESR2 |  |  |
| Salviolone | 11 | PTGS1, CHRM3, CHRM1, DRD5, SCN5A, CHRM5, PTGS2, ADRA2A, HTR3A, CHRM4, OPRD1 |  |  |
| 3-beta-Hydroxymethyllenetanshiquinone | 10 | CHRM1, PTGS2, RXRA, OPRD, ACHE, ADRA1A, ADRB2, OPRM1, PRSS1, NCOA1 |  |  |
| 5,6-dihydroxy-7-isopropyl-1,1-dimethyl-2,3-dihydrophenanthren-4-one | 13 | PTGS1, CHRM3, CHRM1, SCN5A, PTGS2, RXRA, ACHE, ADRA1A, ADRA1B, ADRB2, OPRM1, NCOA2, NCOA1 |  |  |
| 4-methylenemiltirone | 26 | PTGS1, CHRM3, CHRM1, ESR1, AR, SCN5A, PPARG, CHRM5, PTGS2, ADRA2A, ADRA2C, CHRM4, RXRA, OPRD1, ADRA1A, CHRM2, ADRA1B, SLC6A3, ADRB2, ADRA1D, SLC6A4, DRD2, OPRM1, GABRA1, NCOA2, NCOA1 |  |  |
| 1-methyl-8,9-dihydro-7H-naphtho[5,6-g]benzofuran-6,10,11-trione | 12 | PTGS1, CHRM3, SCN5A, CHRM5, PTGS2, RXRA, ACHE, ADRA1A, ADRB2, OPRM1, GABRA1, NCOA1 |  |  |
| C09092 | 10 | CHRM3, CHRM1, SCN5A, ACHE, ADRA1A, CHRM2, ADRA1B, ADRB2, ADRA1D, OPRM1 |  |  |
| Sugiol | 15 | CHRM3, CHRM1, SCN5A, CHRM5, PTGS2, CHRM4, OPRD1, ACHE, ADRA1A, CHRM2, ADRA1B, ADRB2, ADRA1D, DRD2, OPRM1 |  |  |
| Miltipolone | 2 | ESR1, ACHE |  |  |
| Poriferast-5-en-3beta-ol﻿ | 2 | PGR, NCOA2 |  |  |
| Methylenetanshinquinone | 16 | CHRM3, CHRM1, SCN5A, CHRM5, PTGS2, RXRA, OPRD1, ACHE, ADRA1A, CHRM2, ADRB2, SLC6A4, OPRM1, GABRA1, PRSS1, NCOA1 |  |  |
| Przewalskin a | 2 | NR3C2, NR3C1 |  |  |
| Dihydrotanshinlactone | 23 | PTGS1, CHRM3, CHRM1, ESR1, AR, SCN5A, PPARG, CHRM5, PTGS2, HTR3A, RXRA, ACHE, ADRA1A, ADRA1B, SLC6A3, ADRB2, ADRA1D, SLC6A4, OPRM1, GABRA1, GSK3B, PRSS1, CCNA2 |  |  |
| 1,2,5,6-tetrahydrotanshinone | 21 | PTGS1, CHRM3, CHRM1, SCN5A, CHRM5, PTGS2, HTR3A, CHRM4, RXRA, OPRD1, ADRA1A, CHRM2, ADRA1B, SLC6A3, ADRB2, ADRA1D, OPRM1, GABRA1, NCOA2, NCOA1, SLC6A4 |  |  |
| Miltirone | 21 | PTGS1, CHRM3, CHRM1, ESR1, AR, DRD5, SCN5A, CHRM5, PTGS2, ADRA2C, CHRM4, RXRA, OPRD1, ADRA1A, CHRM2, ADRA1B, SLC6A3, ADRB2, ADRA1D, OPRM1, NCOA2 |  |  |
| Dan-shexinkum d | 19 | PTGS1, KCNH2, CHRM1, ESR1, AR, SCN5A, PPARG, PTGS2, RXRA, ACHE, ADRA1B, ADRB2, ESR2, GSK3B, CHEK1, PRSS1, CCNA2, NCOA2, NCOA1 |  |  |
| Neocryptotanshinone ii | 21 | PTGS1, CHRM3, CHRM1, ESR1, AR, SCN5A, PTGS2, CHRM4, RXRA, OPRD1, ADRA1A, CHRM2, ADRA1B, SLC6A3, ADRB2, ADRA1D, SLC6A4, OPRM1, GABRA1, GSK3B, CCNA2 |  |  |
| Przewaquinone f | 3 | PTGS2, PRSS1, NCOA1 |  |  |
| 2-isopropyl-8-methylphenanthrene-3,4-dione | 23 | PTGS1, CHRM3, CHRM1, ESR1, AR, SCN5A, PPARG, CHRM5, PTGS2, HTR3A, CHRM4, RXRA, ADRA1A, CHRM2, ADRA1B, SLC6A3, ADRB2, ADRA1D, SLC6A4, OPRM1, GABRA1, CCNA2, NCOA2 |  |  |
| (6S,7R)-6,7-dihydroxy-1,6-dimethyl-8,9-dihydro-7H-naphtho[8,7-g]benzofuran-10,11-dione | 4 | PTGS2, ACHE, PRSS1, NCOA1 |  |  |
| Tanshindiol B | 3 | PTGS2, ACHE, NCOA1 |  |  |
| Przewaquinone E | 3 | PTGS2, ACHE, NCOA1 |  |  |
| Salvianolic acid j | 2 | F7, PRSS1 |  |  |
| Sclareol | 1 | PTGS2 |  |  |
| Dehydrotanshinone II A | 16 | CHRM3, CHRM1, ESR1, AR, SCN5A, PPARG, CHRM5, PTGS2, CHRM4, OPRD1, ACHE, ADRA1A, ADRB2, OPRM1, GABRA1, NCOA1 |  |  |
| Poriferasterol | 2 | PGR, NR3C2 |  |  |
| 3α-hydroxytanshinoneⅡa | 10 | CHRM1, SCN5A, CHRM5, PTGS2, OPRD1, ACHE, ADRB2, OPRM1, PRSS1, NCOA1 |  |  |
| Manool | 1 | NCOA2 |  |  |
| Isoimperatorin | 1 | PTGS2 |  |  |
| Salvianolic acid g | 1 | PTGS2 |  |  |
| (E)-3-[2-(3,4-dihydroxyphenyl)-7-hydroxy-benzofuran-4-yl]acrylic acid | 1 | PTGS2 |  |  |
| Deoxyneocryptotanshinone | 20 | PTGS1, CHRM3, CHRM1, ESR1, AR, SCN5A, CHRM5, PTGS2, CHRM4, RXRA, OPRD1, ADRA1A, CHRM2, ADRA1B, ADRB2, ADRA1D, OPRM1, GSK3B, NCOA2, NCOA1 |  |  |
| Tanshinone iia | 37 | CHRM3, CHRM1, SCN5A, CHRM5, PTGS2, CHRM4, OPRD1, ACHE, ADRA1A, CHRM2, ADRB2, OPRM1, NCOA1, RXRA, RELA, BCL2, FOS, CDKN1A, MMP9, JUN, AHSA1, CASP3, TP63, NFKBIA, FASN, EDNRA, EDN3, CYP3A4, CYP1A2, MYC, CYP1A1, NR1I2, NPM1, ECE1, PARP4, CALCR, ITGB3 |  |  |
| Isotanshinone II | 19 | CHRM3, CHRM1, ESR1, AR, SCN5A, CHRM5, PTGS2, RXRA, OPRD1, ACHE, ADRA1A, CHRM2, ADRB2, OPRM1, ESR2, GABRA1, GSK3B, CHEK1, CCNA2 |  |  |
| Danshenspiroketallactone | 19 | PTGS1, CHRM3, CHRM1, ESR1, SCN5A, CHRM5, PTGS2, CHRM4, RXRA, ACHE, ADRA1A, CHRM2, ADRA1B, ADRB2, ADRA1D, CHRNA2, SLC6A4, OPRM1, GABRA1 |  |  |
| Cryptotanshinone | 26 | PTGS1, CHRM3, CHRM1, SCN5A, CHRM5, PTGS2, CHRM4, OPRD1, ADRA1A, CHRM2, ADRA1B, ADRB2, ADRA1D, OPRM1, NCOA2, NCOA1, PGR, GABRA1, RELA, STAT3, CCND1, BCL2L1, TNFSF15, APP, EDN3, BIRC5 |  |  |
| Tanshinaldehyde | 8 | CHRM1, PTGS2, OPRD1, ACHE, ADRB2, OPRM, PRSS1, NCOA1 |  |  |
| Neocryptotanshinone | 12 | PTGS1, CHRM3, CHRM1, SCN5A, PPARG, PTGS2, ADRA1B, ADRB2, ADRA1D, OPRM1, NCOA2, NCOA1 |  |  |
| Isocryptotanshi-none | 23 | PTGS1, CHRM3, CHRM1, ESR1, AR, SCN5A, CHRM5, PTGS2, CHRM4, RXRA, OPRD1, ACHE, ADRA1A, CHRM2, ADRA1B, ADRB2, ADRA1D, DRD2, OPRM1, GABRA1, PRSS1, NCOA2, NCOA1 |  |  |
| Przewaquinone c | 15 | PTGS1, CHRM3, CHRM1, SCN5A, CHRM5, PTGS2, CHRM4, OPRD1, ACHE, ADRA1A, CHRM2, ADRB2, OPRM1, GABRA1, NCOA1 |  |  |
| Danshenol A | 6 | PTGS1, KCNH2, SCN5A, PTGS2, RXRA, NCOA1 |  |  |
| Danshenol B | 5 | PTGS2, PGR, OPRM1, NR3C1, NCOA1 |  |  |
| Digallate | 2 | PTGS2, AKR1B1 |  |  |
| Przewaquinone B | 4 | PTGS2, RXRA, PRSS1, NCOA1 |  |  |
| 2-(4-hydroxy-3-methoxyphenyl)-5-(3-hydroxypropyl)-7-methoxy-3-benzofurancarboxaldehyde | 7 | ESR1, AR, PPARG, ESR2, MAPK14, GSK3B, CCNA2 |  |  |
| Prolithospermic acid | 5 | PTGS1, ESR1, AR, PTGS2, PRSS1 |  |  |
| (6S)-6-(hydroxymethyl)-1,6-dimethyl-8,9-dihydro-7H-naphtho[8,7-g]benzofuran-10,11-dione | 10 | CHRM1, SCN5A, PTGS2, OPRD1, ACHE, ADRA1A, ADRB2, OPRM1, PRSS1, NCOA1 |  |  |
| Epidanshenspiroketallactone | 18 | PTGS1, CHRM3, CHRM1, ESR1, SCN5, CHRM5, PTGS2, CHRM4, RXRA, OPRD1, ADRA1A, CHRM2, ADRA1B, ADRB2, ADRA1D, SLC6A4, OPRM1, GABRA1 |  |  |
| Formyltanshinone | 4 | AR, PTGS2, RXRA, NCOA1 |  |  |
| (6S)-6-hydroxy-1-methyl-6-methylol-8,9-dihydro-7H-naphtho[8,7-g]benzofuran-10,11-quinone | 10 | PTGS2, ACHE, PRSS1, NCOA1, ESR1, AR, PPARG, PTGS2, PRSS1, CCNA2 |  |  |
| Przewalskin b | 6 | PTGS2, PGR, NR3C2, NR3C1, NCOA2, NCOA1 |  |  |
| 6,8-Dihydroxy-7- methoxyxanthone | 7 | PTGS1, PTGS2, ADRB2, MAPK14, GSK3B, CHEK1, PKIA |  |  |
| Luteolin | 49 | PTGS1, AR, PTGS2, PRSS1, NCOA2, RELA, EGFR, AKT1, VEGFA, CCND1, BCL2L1, CDKN1A, CASP9, MMP2, MMP9, MAPK1, IL10, RB1, TNFSF15, JUN, IL6, CASP3, TP63, NFKBIA, TOP1, MDM2, APP, MMP1, PCNA, ERBB2, PPARG, HMOX1, CASP7, ICAM1, MCL1, BIRC5, IL2, CCNB1, TYR, IFNG, IL4, TOP2A, GSTP1, INSR, CD40LG, PTGES, NUF, ADCY2, MET |  |  |
| Quercetin | 134 | PTGS1, AR, PPARG, PTGS2, NCOA2, AKR1B1, PRSS1, KCNH2, SCN5A, ADRB2, MMP3, F7, RXRA, ACHE,GABRA1, MAOB, RELA, VEGFA, CCND1, BCL2, BCL2L1, FOS, CDKN1A, EIF6, BAX, CASP9, PLAU, MMP2, MMP9, MAPK1, IL10, EGF, RB1, TNFSF15, JUN, IL6, AHSA1, CASP3, TP63, ELK1, NFKBIA, POR,ODC1, CASP8, TOP1, RAF1, SOD1, PRKCA, MMP1, HIF1A, STAT1, RUNX1T1, ERBB2, PPARG, ACACA, HMOX1, CYP3A4, CYP1A2, CAV1, MYC, F3,GJA1,CYP1A1, ICAM1, IL1B, CCL2, SELE, VCAM1, PTGER3, BIRC5, DUOX2, HSPB1, SULT1E1, IL2, NR1I2, CYP1B1, CCNB1, PLAT, THBD, SERPINE1, COL1A1, IFNG, ALOX5, IL1A, MPO, TOP2A, NCF1, ABCG2, HAS2, GSTP1, NFE2L2, NQO1, PARP1, AHR, PSMD3, COL3A1, CXCL11, CXCL2, DCAF5, NR1I3, CHEK2, INSR, CLDN4, PPARA, PPARD, HSF1, CRP, CXCL10, CHUK, SPP1, RUNX2, RASSF1, CXCL2, DCAF5, NR1I3, CHEK2, INSR, CLDN4, PPARA, PPARD, HSF1, CRP, CXCL10, CHUK, SPP1, RUNX2, RASSF1, RASA1, GSTM1, GSTM2, PRKCB, CXCL8, AKT1, EGFR |  |  |
| Rhein | 5 | PTGS1, PTGS2, NCOA2, AKR1B1, JUN |  |  |
| (+)-catechin | 7 | PTGS1, ESR1, PTGS2, NCOA2, RXRA, CAT, HAS2 |  |  |
| Picralinal | 4 | AR, SCN5A, OPRD1, OPRM1 |  |  |
| Physovenine | 26 | PTGS1, CHRM3, CHRM1, ESR1, AR, SCN5A, PTGS2, RXRA, OPRD1, ACHE, SLC6A2, ADRA1A, CHRM2, ADRA2B, ADRA1B, SLC6A3, ADRB2, CHRNA2, SLC6A4, OPRM1, ESR2, GABRA1, GSK3B, PRSS1, CCNA2, GRIA2 |  |  |
| Stigmasterol | 26 | PGR, NR3C2, NCOA2, ADH1C, RXRA, NCOA1, PTGS1, PTGS2, ADRA2A,  SLC6A2, SLC6A3, ADRB2, AKR1B1, PLAU, LTA4H, MAOB, MAOA, CTRB1,  CHRM3, CHRM1, ADRB1, SCN5A, ADRA1A, CHRM2, ADRA1B, GABRA1 |  | PTGS1, PTGS2, NCOA2 |
| Myricanone | 17 | PTGS1, KCNH2, ESR, AR, SCN5A, PPARG, PTGS2, F7, KDR, RXRA, ADRB2, ESR2, MAPK14, GSK3B, CHEK1, CCNA2, NCOA1 |  |  |
| Perlolyrine | 2 | PTGS2, RXRA |  |  |
| Wallichilide﻿ | 4 | PTGS2, NR3C2, NR3C1, NCOA2 |  |  |
| Sitosterol | 3 | PGR, NCOA2, NR3C2 |  |  |
| FA | 1 | GSK3B |  |  |
| Hederagenin | 16 | PGR, NCOA2, CHRM3, CHRM1, CHRM2, ADRA1B, GABRA1, GRIA2, ADH1B, ADH1C, LYZ, PTGS1, SCN5A, PTGS2, RXRA, SLC6A2 |  |  |
| Campesterol | 4 | PGR, PTGS1, PTGS2, NCOA2 |  |  |
| 3-O-p-coumaroylquinic acid | 3 | PTGS1, PTGS2, NCOA2 |  |  |
| Sitosterol alpha1 | 5 | PGR, PTGS2, GABRA, ADH1C, NR3C2 |  |  |
| 2,3-didehydro GA70 | 7 | PTGS1, CHRM1, PTGS2, SLC6A2, GABRA1, PRSS1, GRIA2 |  |  |
| GA54 | 2 | PTGS2, NCOA2 |  |  |
| GA63﻿ | 3 | PTGS2, GABRA1, GRIA2 |  |  |
| GA87 | 1 | PTGS2 |  |  |
| GA121-isolactone | 1 | PGR |  |  |
| Gibberellin 7 | 6 | CHRM3, CHRM1, PTGS2, SLC6A, ADRB2, SLC6A4 |  |  |
| GA120 | 5 | CHRM3, CHRM1, PTGS2, CHRM2, GABRA1 |  |  |
| GA77 | 2 | GABRA1, GRIA2 |  |  |
| 2,3-didehydro GA77 | 3 | PTGS2, GABRA1, NCOA2 |  |  |
| GA122-isolactone | 1 | PGR |  |  |
| 4a-formyl-7alpha-hydroxy-1-methyl-8-methylidene-4aalpha,4bbeta-gibbane-1alpha,10beta-dicarboxylic acid | 2 | NR3C2, PGR |  |  |
| GA60 | 3 | CHRM2, GABRA1, GRIA2 |  |  |
| Gibberellin A44 | 2 | NR3C2, GABRA1 |  |  |
| 16beta,17-dihydroxy-(-)-kauran-19-ate-beta-D-glucose ester_qt | 2 | NR3C2, NCOA2 |  |  |
| Rabdosinatol | 1 | NR3C2 |  |  |
| Shionone | 1 | PGR |  |  |
| Galangin | 10 | PTGS1, AR, PPARG, PTGS2, CHEK1, BCL2, CYP1A1, CCND3, GSTP1, AHR |  |  |
| ZINC03978781 | 3 | PGR, NCOA2, NR3C2 |  |  |
| Kaempferol | 49 | PTGS1, AR, PPARG, PTGS2, NCOA2, PRSS1, PGR, CHRM1, ACHE, SLC6A2, CHRM2, ADRA1B, GABRA1, F7, RELA, IKBKB, AKT1, BCL2, BAX, TNFSF15, JUN, AHSA1, CASP3, MAPK8, MMP1, STAT1, PPARG, HMOX1, CYP3A4, CYP1A2, CYP1A1, ICAM1, SELE, VCAM1, NR1I2, CYP1B1, ALOX5, HAS2, GSTP1, AHR, PSMD3, NR1I3, INSR, DIO1, PPP3CA, GSTM1, GSTM2, AKR1C3, SLPI |  |  |
| Spinasterol | 3 | PGR, NR3C2, NCOA2 |  |  |
| 5,7-dihydroxy-2-(3-hydroxy-4-methoxyphenyl)chroman-4-one | 5 | PTGS1, PTGS2, SCN5A, NCOA2, NCOA1 |  |  |
| Xanthinin | 98 | FNTA, ACHE, PRKCA, CYP19A1, GRM5, HTR2B, FAP, CTSK, BCL2L1,  P2RX7, PDE4D, CDC25B, BRD4, MTNR1B, PTGS2, MTNR1A, IL1B, MAOB, CAPN1, NQO2, MAPK8, MALT1, TAAR1, CSF1R, TGM2, SIRT2, PSEN2, PTPN1, CDK5R1, CTSS, CTSB, ALPL, POLA1, POLB, JAK3, JAK1, JAK2, PARP1, TYK2, IKBKB, TLR9, KCNJ5, KCNJ6, CDC25A, AR, GABRB3, JAK3, CACNA1B, CXCR3, ECE1, AKR1B1, GSK3A, MME, CDC25C, HSD11B1, DPP4, CCND2, CTSL, GLP1R, PRKD1, HCRTR2, HCRTR1, KDM4C, ABCG2, NOS1, NAAA, S1PR2, F10, MAPK14, TTR, PDE10A, SIGMAR1, MMP2, IRAK4, RIPK1, GRK6, IDH1, ADORA2A, MMP3, MMP9, MMP1, CREBBP, CYP11B1, EPHX2, CYP11B2, SIRT1, KDM1A, CDK2, CFD, F13A1, PPARA, PPARD, FLT1, PDGFRB, CCNB3,  CCND1, CDK6, PYGL |  |  |
| Xanthine | 23 | RORC, ATP1A1, STAT3, PRKCA, GLRA1, GLRA2, PTGS2, F2RL1, PPM1B,  PPP2R5A, CASP3, CASP7, PRKCE, PRKCH, PRKCQ, PTPN1, PLA2G1B, GLI1, PPP2CA, CTSD, AR, JUN, PPP1CC |  |  |
| Hyrcanoside | 23 | RORC, ATP1A1, STAT3, PRKCA, GLRA1, GLRA2, PTGS2, F2RL1, PPM1B,  PPP2R5A, CASP3, CASP7, PRKCE, PRKCH, PRKCQ, PTPN1, PLA2G1B, GLI1, PPP2CA, CTSD, AR, JUN, PPP1CC |  |  |
| Guanine(1,7-dihydro-form) | 4 | PNP, GDA, TK1, ACHE |  |  |
| Cholesteryl ferulate | 20 | EDNRA, POLB, BACE1, PDE9A, PDE1B, MET, ELANE, ACP1, HDAC3, HDAC6, HDAC2, HDAC1, HDAC8, HDAC11, HDAC4,  DYRK1A, CLK1, PRKCA, DYRK2, DYRK1B |  |  |
| Cholesterol | 36 | AR, ESR1, ESR2, CYP17A1, CYP19A1, NR1H3, SREBF2, NPC1L1, HMGCR, RORC, CYP51A1, SHBG, PTPN1, SERPINA6, G6PD, CHRM2,  CNR1, CYP2C19, GCGR, ACHE, SLC6A2, SLC6A4, KMT5A, SQLE, PSEN2, PRKCG, PRKCD, PRKCB, PRKCE, PRKCQ, OXTR, TACR1, CCR1, SLC6A9, CHRM5, HSD17B1 |  |  |
| 4-guanidino-1-butanol | 5 | ADRB2, PNP, FUCA1, GBA, PLG |  |  |
| Siegesmethyletheric acid | 3 | PGR, NR3C2, NCOA2 |  |  |
| Siegesesteric acid II | 3 | PGR, NR3C2, NCOA2 |  |  |
| Coronaridine | 19 | PTGS1, CHRM3, CHRM1, DRD5, SCN5A, CHRM5, HTR3A, CHRM4, OPRD1, ADRA1A, ADRA2B, ADRA1B, ADRB2, ADRA1D, CHRNA2, SLC6A4, DRD2, OPRM1, GABRA1 |  |  |
| Vernolic acid | 1 | PTGS2 |  |  |
| 15alpha-Hydroxy-ent-kaur-16-en-19-oic acid | 3 | PGR, NR3C2, NCOA2 |  |  |
| (1R)-1-[(2S,4aR,4bS,7R,8aS)-7-hydroxy-2,4b,8,8-tetramethyl-4,4a,5,6,7,8a,9,10-octahydro-3H-phenanthren-2-yl]ethane-1,2-diol | 3 | PGR, NCOA2, NCOA1 |  |  |
| Heptyl phthalate | 2 | SLC6A2, ADRB2 |  |  |
| (3S,6S)-3-(benzyl)-6-(4-hydroxybenzyl)piperazine-2,5-quinone | 3 | AR, PTGS2, ADRB2 |  |  |
| Beta-D-Ribofuranoside,  xanthine-9 | 2 | MTAP, PTGS2 |  |  |
| 10,13-eicosadienoic | 2 | PTGS1, NCOA2 |  |  |
| Cycloartenol | 1 | NR3C2 |  |  |
| 24-Ethylcholest-4-en-3-one | 2 | PGR, NR3C2 |  |  |
| Cavidine | 20 | PTGS1, CHRM3, KCNH2, CHRM1, ADRB1, SCN5A, CHRM5, PTGS2, HTR3A, ADRA2C, CHRM4, RXRA, OPRD1, ADRA1B, ADRB2, ADRA1D, OPRM1, RXRB, SLC6A4, F7 |  |  |
| Baicalein | 27 | PTGS1, AR, PTGS2, PRSS1, NCOA2, NCOA1, RELA, AKT1, VEGFA, BCL2, FOS, BAX, MMP9, CASP3, TP63, HIF1A, FOSL1, FOSL2, CCNB1, MPO, AHR, IGF2, CYCS, TDRD7, EGLN1, NOX5, APOD |  |  |
| Coniferin | 14 | CHRM3, CHRM1, ESR1, AR, SCN5A, PPARG, PTGS2, ADRA1B, ADRB2,  ADRA1D, OPRM1, CCNA2, NCOA2, NCOA1 |  |  |
| Gondoic acid | 2 | PTGS1, NCOA2 |  |  |
| 7beta-(3-ethyl-cis-crotonoyloxy)-14-  hydroxy-notonipetranone | 3 | PTGS2, NCOA2, NCOA1 |  |  |
| 7beta-(4-methylsenecioyloxy) oplopa-3(14)E,8(10)-dien-2-one | 3 | PTGS2, RXRA, NCOA2 |  |  |
| Femara | 2 | PTGS1, PTGS2 |  |  |
| Tussilagin | 4 | PTGS2, NR3C2, NCOA2, NCOA1 |  |  |
| Senkirkine | 2 | PTGS2, NCOA2 |  |  |
| 14-acetoxy-7beta-angeloyloxy-notonipetranone | 4 | PTGS2, NR3C2, NCOA2, NCOA1 |  |  |
| 7beta-(3-ethyl-ciscrotonoyloxy)-la- (2-methyl butyryloxy)-3,14-dehydro-Z-notonipetralactone | 1 | NCOA2 |  |  |
| Methyl 3-o-caffeoylquinate | 1 | PTGS2 |  |  |
| La-7beta-di(4-methulsenecioyloxy) oplopa-3(14)Z,8(10)-dien-2-one | 2 | NR3C1, NCOA2 |  |  |
| 7beta-angeloyloxyoplopa- 3(14)Z,8(10)-dien-2-one | 3 | PTGS2, NCOA2, NCOA1 |  |  |
| 14-acetoxy-7beta-senecioyloxy-notonipetranone | 3 | NR3C2, NCOA2, NCOA1 |  |  |
| Beta-sitosterol | 26 | PGR, NCOA2, PTGS1, PTGS2, KCNH2, CHRM3, CHRM1, SCN5A, CHRM4, ADRA1A, CHRM2, ADRA1B, ADRB2, CHRNA2, SLC6A4, OPRM1, GABRA1, BCL2, BAX, CASP9, JUN, CASP3, CASP8, PRKCA, PON1, MAP2 |  |  |
| Methyl butyric acid tussilagin ester | 1 | NCOA2 |  |  |
| 7beta-senecioyloxyoplopa-3(14)Z,8(10)-dien-2-one | 3 | NR3C2, NR3C1, NCOA2 |  |  |

Supplementary Table 2: The table of idiopathic pulmonary fibrosis related targets.

| **Gene name** |  |  |  |
| --- | --- | --- | --- |
| TERT | SFTPD | VEGFA | TNNT2 |
| RTEL1 | DSP | ACVRL1 | HLA-B |
| CFTR | FAM13A | NKX2-5 | CXCL10 |
| SFTPC | CTNNB1 | CTLA4 | FGF2 |
| ABCA3 | TGFBR1 | FAM111B | CXCR3 |
| TNF | CXCL8 | MMP9 | JAG1 |
| IL6 | NHP2 | PPARG | FGFR1 |
| TGFB1 | FASLG | STAT3 | CCL3 |
| MUC5B | NOP10 | IL17A | IL1A |
| SFTPA2 | STN1 | SERPINC1 | SMAD9 |
| SFTPB | TGFB2 | TIMP1 | NOS3 |
| IL10 | AGT | BMP6 | CLCN2 |
| PARN | DKC1 | REN | SCNN1A |
| SFTPA1 | COL1A2 | HLA-DQB1 | FN1 |
| RTEL1-TNFRSF6B | F2 | FOXF1 | STAT4 |
| TERC | ACTC1 | HFE | IL1R1 |
| IFNG | ATP11A | FAS | HGF |
| ACE | IL1B | GATA4 | HLA-DPB1 |
| BMPR2 | SERPINH1 | IGF1 | PTPN22 |
| ELN | DPP9 | TTN | THBD |
| CAV1 | ALOX5 | FBN1 | MUC5AC |
| TLR4 | EGF | AGTR1 | SPP1 |
| CCN2 | WRAP53 | CSF2 | CC2D2A |
| IL13 | EDN1 | MBL2 | LMNA |
| SERPINE1 | PRTN3 | PDGFRA | SMPD1 |
| HLA-DRB1 | ALB | IL5 | NPPB |
| SERPINA1 | COL3A1 | FCGR2A | KCNQ1 |
| MUC1 | CRP | MIF | HIF1A |
| CCR6 | MIR130A | CD4 | CD40LG |
| MMP1 | ENG | LEP | HLA-DQA1 |
| MIR21 | HMOX1 | CCL11 | THPO |
| IL1RN | CCL2 | SRC | CXCR4 |
| TP53 | MIRLET7D | NKX2-1 | HPS1 |
| SMAD3 | PDGFRB | IL18 | SLC20A2 |
| PDGFB | ELANE | TNFRSF1A | NOTCH1 |
| MMP2 | MIR34C | TMEM67 | SCGB1A1 |
| SMAD4 | IL4 | WT1 | KIT |
| AKT1 | KCNK3 | TLR2 | KRT18 |
| TINF2 | ADRB2 | CCL5 | TNNI3 |
| COL1A1 | IL2RA | MPO | EGFR |
| ELMOD2 | IL33 | CXCL1 | GJA1 |
| JAK2 | GREM1 | IL2 | ATP12A |
| CCL18 | CCR4 | GSN | IGFBP5 |
| CCL17 | TLR3 | SPPL2C | ACTB |
| TLR9 | ADCY10 | MIR30A | PTEN |
| MMP7 | POSTN | MYH6 | IL2RB |
| KIF7 | TGFB3 | APOE | MIR214 |
| VCAM1 | PTPRC | HRAS | BMP2 |
| SCN5A | MIR199A1 | DDR1 | RNASE3 |
| ITGAM | FLNC | GAPDH | PMS2 |
| NOD2 | MIR17 | HPS5 | MAPT |
| MTHFR | CSF2RA | KITLG | NLRC4 |
| FLNA | DMD | HPS3 | NRAS |
| PTPN11 | MAPK8 | PDGFA | S100A1 |
| HPS4 | ZCCHC8 | MIR223 | THY1 |
| AGER | CP | TRPC6 | CCN4 |
| SMAD2 | LPAR1 | HPS6 | GLI3 |
| RPL5 | DES | S100A4 | TEK |
| RYR1 | SOS1 | ABCB11 | MIR126 |
| CHD7 | LOC110806263 | SMAD7 | MIR140 |
| ADA | INS | TSLP | TBX20 |
| IL9 | CXCL5 | LOXL2 | IL12B |
| LOX | CCR7 | CR1 | PRSS1 |
| NOX4 | SCNN1G | PKD2 | SNAI1 |
| MMP12 | CXCR2 | IQCB1 | ATP4A |
| CD34 | IFT43 | GJA5 | CPS1 |
| GDF1 | SCNN1B | EIF2AK4 | CD19 |
| CASR | PKD1 | TNFRSF11B | GATA2 |
| ICOSLG | NF1 | ICAM1 | LOC111674472 |
| ACTA2 | TOLLIP | INPP5E | MIR155 |
| TGFBR2 | CDH1 | ALMS1 | STAT6 |
| CCL4 | MYH7 | TNFRSF13B | APOB |
| CXCR1 | SPINK1 | CLCN5 | GBA |
| ACP5 | ANKRD1 | FHIT | FOS |
| CACNA1H | SPARC | BTNL2 | KCNH2 |
| CD8A | HSPG2 | CALR | MIR204 |
| CXCL9 | CCL22 | CSF3 | TBX1 |
| CXCL12 | GRP | ACD | MEG3 |
| CHI3L1 | STAT1 | ENPP2 | TNFSF11 |
| HMGB1 | GNRH1 | RPGRIP1L | NPPA |
| FGF7 | KRAS | MIR142 | PRDM10 |
| MPL | SLC2A1 | AGTR2 | ACVR1 |
| MT-CO1 | IRF5 | CCR2 | SERPINA3 |
| RYR2 | LBR | GCH1 | CTSG |
| LAMP2 | CASP8 | PRKAG2 | KCNJ2 |
| TRPV4 | CD36 | IGF2 | ATM |
| F3 | EPO | COPA | GDF2 |
| VWF | NFKB1 | SLPI | BGLAP |
| NPHS1 | PIK3CA | GPT | MB |
| GATA6 | CALCA | SOD1 | PLG |
| NOS2 | MYBPC3 | NEB | ZEB2 |
| MIR144 | ADAMTS13 | ADM | TPM2 |
| SELP | MIR29A | VDR | PRKD1 |
| LAMA2 | CCR5 | FKRP | IGF2R |
| GAA | HP | NRXN1 | KDR |
| SLC6A4 | PAX2 | SCARB2 | POLG |
| RAF1 | MIR22 | HARS1 | BMP1 |
| F13A1 | TXNRD2 | SLC17A5 | HBB |
| MIR338 | S100A9 | TNFRSF1B | CACNA1D |
| RET | ABCC2 | FGFR2 | GNAS |
| NFE2L2 | CSF3R | LTA | ASXL1 |
| TPM1 | PROC | GSTP1 | MALAT1 |
| ACTA1 | ABCC9 | EDNRB | NPM1 |
| MIR409 | FOXP3 | WDR11 | THBS1 |
| CD2AP | CCND1 | ERBB2 | ACTN2 |
| MIR708 | CACNA1C | SDHA | PGM1 |
| MIR146A | FGFR3 | ERCC6 | TSC2 |
| EDNRA | GSTM1 | LGALS3 | IL15 |
| TYR | IL12RB1 | BBIP1 | SELE |
| PKHD1 | MMP3 | MT-ND1 | SHH |
| MYPN | BDNF | PLAT | H19 |
| IFNA1 | UMOD | RBM20 | MIR20A |
| NPHS2 | ITGA3 | CASP3 | PIK3C2A |
| TET2 | ARHGAP31 | BAG3 | COMT |
| ATP8B1 | FARSB | LPL | CDKN1A |
| TBX5 | PTGS2 | ABCB1 | CD80 |
| VIP | INVS | GABRA1 | NOS1 |
| H2AC18 | TLR5 | HLA-A | COL4A3 |
| NPHP4 | RSPO2 | SLC34A1 | IL3 |
| TBX4 | JUN | VCL | BOLA3 |
| HLA-DPA1 | PDE5A | FMR1 | WRN |
| PRKN | S100A8 | FKTN | CEP104 |
| LRBA | SLC34A2 | TCAP | MIR221 |
| IL12A | CAT | HAMP | SHOX |
| F5 | ADIPOQ | MTOR | PF4 |
| MIR34A | EPHX1 | NFU1 | MAPK7 |
| TRIM21 | FBL | MIR27A | PKP2 |
| SOD2 | HSPA4 | PRL | F2R |
| DNAH8 | ICOS | LAMA4 | CSRP3 |
| DKK1 | AGL | CDC42 | FHL2 |
| MAPK14 | CD79A | DYNC2H1 | COL2A1 |
| MYL2 | MMP8 | IFNGR1 | PTX3 |
| TNNC1 | VCP | PRDM16 | CMA1 |
| SOD3 | IDH1 | PRKACA | TCIRG1 |
| NUP107 | PPARA | TAC1 | GHR |
| MT-TL1 | IFNA2 | KNG1 | SDCCAG8 |
| BMP4 | IL13RA2 | MIR145 | PPCS |
| MYOCD | CDKN1B | HMGCR | SLC40A1 |
| CAV3 | JPH2 | FADD | ABCC1 |
| KCNJ5 | SETD2 | ANGPT1 | SOX9 |
| WASHC5 | ZMPSTE24 | SELENON | MIR200B |
| PLOD2 | CYP2E1 | BMP7 | STX1A |
| APOH | COL4A5 | HCN4 | ANK2 |
| SDHB | SGCD | NAGLU | SMARCAL1 |
| MYLK | NLRP3 | PRKCD | CD28 |
| SOX10 | MIR222 | PLA2G7 | NR3C2 |
| DEFB1 | SBDS | BPI | VPS45 |
| KRT7 | C4A | AKAP9 | HJV |
| IGHE | NR3C1 | GZMB | AP3D1 |
| CYP1A1 | MT-CYB | LTF | PSMD4 |
| NIPBL | LRP2 | DOLK | RUNX1 |
| IGFBP3 | PDE4A | LDB3 | RHOA |
| CCR3 | VEGFC | PLEC | CHAT |
| SST | MYL3 | INSR | ITGA7 |
| CHIT1 | MT-CO3 | CACNB2 | AFF4 |
| SLC11A1 | MET | ACTG2 | CASP10 |
| ZNF423 | MAPK1 | TNNI2 | MEFV |
| TRAF3IP1 | CSF1 | WNT1 | TGFA |
| IDH2 | TNFSF13B | TMPO | PTH |
| KCNJ8 | STING1 | MUSK | PLA2G6 |
| GGT1 | SH2B3 | TTR | PDCD1 |
| VIM | TF | ABCB4 | HSPD1 |
| TAZ | TPM3 | SRA1 | TIMP2 |
| ENPP1 | GUSB | DSG2 | ALPL |
| CRYAB | MYC | GALC | MIR200C |
| IFIH1 | GATAD1 | SHOC2 | DCN |
| NR1H4 | ITGB3 | CDKN3 | PLAU |
| MT-ATP6 | GC | BRAF | SNCA |
| PLN | PRF1 | CYCS | CD86 |
| CTC1 | EPRS1 | SGK1 | CYP3A4 |
| FIP1L1 | TSC1 | GFI1B | BSCL2 |
| CDKN1C | PNPLA6 | KCNMA1 | MT-ND5 |
| PSMB8 | SERPINB1 | MRAS | CERS1 |
| AFP | S100A12 | GAST | HMGA2 |
| NPC1 | TNC | TNFRSF6B | EZH2 |
| COL11A2 | U2AF1 | CD44 | NUP133 |
| AGPAT2 | LOC110806306 | PSMC4 | CD27 |
| BCL2 | PHOX2B | MT-ND4 | BBS10 |
| HDAC9 | CD69 | IRF1 | MECP2 |
| MGP | UTP4 | AARS1 | MLXIPL |
| TFR2 | TUBB3 | GLA | ITGB1 |
| ADA2 | KCNJ1 | SOCS3 | NOTCH2 |
| NEXN | FAN1 | GPD1L | CPLANE1 |
| NPC2 | NCF2 | MT-ND6 | FLT1 |
| IKBKG | GSTT1 | EYA4 | MDM2 |
| SOX3 | CTSL | HDAC2 | ANXA5 |
| SIRT1 | TRPM4 | RSPH9 | POMC |
| EPX | ADORA1 | LEPR | BMPR1A |
| HAND2 | SLC9A3 | HLA-G | VAC14 |
| SYP | DEFB4A | ESR1 | TRPV1 |
| MAPK3 | ERF | IL6R | FIG4 |
| PROS1 | MYH14 | LZTR1 | BCL2L1 |
| CACNA2D1 | NEBL | MIR195 | BBS12 |
| RBP4 | SRSF2 | G6PD | PAH |
| RAD21 | TNFSF10 | RETN | NUP85 |
| SLC4A1 | PRODH | LAMB2 | NUP160 |
| HSPA8 | LARS2 | CEP83 | WNT7B |
| HNMT | RPS27A | NR5A1 | SCN1B |
| ITGA2B | IL23R | PROKR2 | ARG1 |
| SOX18 | MIR143 | VDAC1 | ADRB1 |
| ADORA2B | KDM6A | HBA2 | CST3 |
| IL4R | SERPINF2 | NR0B1 | CD63 |
| F8 | NFKB2 | BAX | TOP1 |
| CRB2 | SLCO2A1 | GPD1 | MME |
| C3 | CHRM3 | NAT2 | MT-TK |
| IL11 | GSR | SDHD | CAPN3 |
| SETBP1 | KCNN4 | TPO | FLT4 |
| DTNBP1 | CFH | NPY | WNT3A |
| MYH11 | MIR196A2 | FSHR | MTUS1 |
| VTN | FGF23 | ANGPT2 | RELA |
| GP1BA | HSP90AA1 | TAC3 | SGO1 |
| STAT5B | RAG2 | MIR192 | PARP1 |
| CASP9 | DNAJC5 | NARS1 | RAB27A |
| TLR1 | TALDO1 | FLT3 | MTUS2 |
| GHRL | MUC6 | MYH9 | AVP |
| IFNB1 | ACTN4 | ACHE | NBAS |
| SCN1A | APP | SGCB | CD274 |
| MYO5A | SNRNP70 | STAT5A | ADORA2A |
| CD40 | CYP2C9 | GRIN2A | TGM2 |
| AR | HTR2B | CNTNAP2 | FSHB |
| GNRHR | TYMP | JAK1 | CCR8 |
| LAMP1 | AQP2 | GDF15 | HYDIN |
| DYSF | SLC12A6 | STH | MIR210 |
| IREB2 | GP6 | CREB1 | ITGAL |
| F9 | TFRC | BGN | APC |
| WNT4 | TNNT1 | SS3 | HCRT |
| MIR191 | F2RL1 | APPL1 | DAG1 |
| PPARGC1A | SHBG | HABP2 | IL17F |
| KRT19 | MIR181A1 | MIR499A | FTL |
| PTGS1 | RAG1 | CR2 | ITGA2 |
| KIF20A | NOTCH3 | PECAM1 | CPT2 |
| ITGAV | PSMB5 | UTS2 | PCDH19 |
| TP63 | RUNX2 | AREG | SCN3A |
| PITX2 | TLR6 | KATNIP | TRIM33 |
| MIR125A | VANGL1 | LBP | DNASE1 |
| HTR2A | CXCL2 | CCBE1 | ILK |
| CSF2RB | CYP11B2 | ACTG1 | TPMT |
| TNFSF15 | APOA1 | EP300 | CDH2 |
| CANX | JRK | TIMP3 | MIR132 |
| IGF1R | TERF1 | PRG2 | GATA3 |
| KIF1C | SEMA3A | LRP1B | FANCA |
| CASP1 | PAM16 | C5 | NR0B2 |
| TARS1 | CD68 | TRPC1 | TH |
| ENO1 | PROK2 | BMP15 | XBP1 |
| KRT8 | ITGA4 | PKD1L1 | MIR423 |
| FCGR3B | APEX1 | GH1 | BTK |
| EDN3 | KCNQ1OT1 | SRY | CD55 |
| CD14 | ALDH2 | CYP2D6 | HLA-C |
| MIR133B | IL7R | DARS2 | TWNK |
| PPBP | PRSS8 | GATA1 | MIR451A |
| CD247 | UNC119 | XPNPEP3 | BRCA2 |
| CADM1 | SOCS1 | MIR29C | PTPRO |
| SDC1 | NQO1 | SYK | EXOSC10 |
| PLAUR | PLA2R1 | CS | SLC34A3 |
| ABL1 | HSPA1A | HBG2 | ATF6 |
| ACADVL | ADK | APELA | COMP |
| STXBP1 | IGES | IL1RAPL2 | KIF1B |
| JAK3 | MYOT | IL7 | UBE2L3 |
| GARS1 | OGG1 | KCNJ11 | PES1 |
| BBS9 | HADHA | GGTLC3 | SLC8A1 |
| HOXD13 | EPOR | SIRT3 | KDM4C |
| CCL26 | TERF2 | GDNF | MT-TH |
| SEMA3E | NR1I3 | MLN | FLNB |
| TNFAIP3 | DYNLT1 | BMPR1B | GALNT3 |
| HUWE1 | IKBKB | XDH | IL12RB2 |
| TNNI3K | B2M | P2RX7 | ABCA1 |
| JUP | FCER2 | IL6ST | IL36G |
| ARSA | MIR146B | AQP1 | CALB2 |
| HSPA5 | WNT3 | C1S | ADAM33 |
| MEN1 | FHL1 | PLA2G1B | AFF2 |
| DNMT3B | CHGA | MRAP | FCGR3A |
| CAVIN1 | SELL | SERPINF1 | IRS1 |
| ATP2A2 | CYBB | CYBA | CA4 |
| ITGA5 | CD1C | LYN | P2RY2 |
| CABIN1 | CTSD | VHL | GGT2 |
| EZR | PTK2 | AXL | COQ8B |
| PDLIM1 | NTS | ANPEP | MMADHC |
| FGA | ALPP | IHH | MIR483 |
| ADH1C | NCF1 | RAB8A | TUBB4A |
| GPC3 | ERAP1 | CCNA2 | TRMU |
| ADGRV1 | ENTPD1 | KCNE2 | PSMC6 |
| MEF2C | LCN2 | HRH2 | PTGER4 |
| TNFRSF10A | PTH1R | RHOD | NT5C1A |
| NAMPT | TLR10 | GNAQ | PSMC3IP |
| SMAD1 | TGM1 | TTN-AS1 | ALK |
| TNFRSF11A | ANXA1 | FAM20C | TCF4 |
| TLR7 | ASCL1 | ADCY3 | EMD |
| RPS19 | MMEL1 | FABP3 | BECN1 |
| USB1 | MTMR10 | AIRE | PLCG2 |
| ASL | GRIN2B | CYSLTR1 | ADAMTSL1 |
| PRKG1 | KANSL1 | MRC1 | BCR |
| WG | SGCA | DHCR24 | GCG |
| ADCY6 | MIR185 | RAP1A | KCNE1 |
| MYBPC1 | NEU1 | PLCE1 | POT1 |
| CD177 | SCN9A | SETX | TCF7 |
| KCND3 | DNMT3A | PTRH2 | ITGA1 |
| CDKN2A | GFAP | MYD88 | KLRK1 |
| NPPC | ADRB3 | SRP72 | PON1 |
| SLC26A9 | KRT13 | ADIPOR2 | XRCC1 |
| TRAM2 | TBCB | DDR2 | MIR124-1 |
| SCO2 | SLC4A4 | MX1 | PDLIM3 |
| PODXL | F10 | DNAAF1 | TRPC3 |
| UCHL1 | RASGRP1 | DPP4 | CD81 |
| INHA | ADGRG6 | NCAM1 | ABCB7 |
| ADCY8 | IL36B | CSN3 | HAVCR1 |
| ACTN1 | CASQ2 | WDTC1 | HBA1 |
| TNFRSF13C | E2F1 | CFL2 | CCK |
| CSF1R | MORC3 | SNAPIN | ABCC6 |
| HPRT1 | PCNT | CCL20 | HTR1B |
| MYOZ2 | ALG6 | ACE2 | ETV6 |
| PNPLA3 | CTSB | MKI67 | CCR1 |
| PTGIR | SCT | MIR125B1 | CETP |
| MIR148A | EPAS1 | MIR183 | PGR |
| GBE1 | CHUK | TAP2 | IL32 |
| DEFB103B | CD163 | SLC2A4 | HSPB1 |
| CTNNA3 | LPA | PIK3CG | DSC2 |
| CLCNKB | DLL3 | LRP5 | ATP2A1 |
| WASHC4 | CRH | GHSR | SLC25A4 |
| PAPPA | FTH1 | UBC | APOC3 |
| ETS1 | SLC12A1 | PUS1 | FHOD3 |
| KL | CGA | NFKBIA | CD209 |
| PMPCA | IFRD1 | SYNPO | PRKG2 |
| IFI16 | RAC1 | PRSS2 | SLC4A5 |
| HTR3A | TMEM43 | ENO2 | MEF2A |
| COL4A4 | IRF3 | FANCD2 | ADAR |
| TPH1 | HOTAIR | SPG7 | F12 |
| PLA2G2A | SUN2 | IL27 | LCAT |
| CDKN2B-AS1 | IL16 | IL36RN | PDE4D |
| HEY2 | TNNI1 | C3orf35 | KEAP1 |
| CYP3A5 | HRC | FCGR2B | XIAP |
| FABP4 | CDK6 | CYP27B1 | TG |
| UGT1A1 | HSPH1 | CRYAA | IL1RL1 |
| APOL1 | MYBPC2 | CASP7 | TPM4 |
| POMT1 | GAR1 | RNF5 | LMX1B |
| XK | OXT | IFNGR2 | MIR25 |
| CD1A | COL4A1 | SERPINA6 | CTF1 |
| SMARCA4 | MBTPS2 | SPINK5 | FBLN5 |
| CXCL13 | NFE2 | NDUFAF6 | MICA |
| GAP43 | CTNNA1 | CACNA1S | CD38 |
| AIF1 | LIG4 | ATXN10 | GRN |
| SGCG | CDX2 | PSMD14 | CDH5 |
| EGR1 | LDLR | CACNB1 | SI |
| CXCR6 | ADIPOR1 | MHRT | HLA-DOA |
| RAB11B | FOXM1 | CHRNA7 | SP1 |
| MIR9-1 | LTBP2 | NPR3 | CD1B |
| PAX6 | MKRN3 | LMOD1 | RPL35A |
| MIR26A1 | ADAM17 | TWIST1 | CCL21 |
| FST | ITGB4 | ESR2 | MUC2 |
| PSMA6 | SLC10A2 | DLK1 | RPS26 |
| ABO | CDK5 | ENPP3 | ELOVL4 |
| MIR212 | LRP6 | CCKBR | MAPT-AS1 |
| ITGB6 | BRD2 | SEMA6A | DHCR7 |
| FGF1 | MIR24-1 | PLP1 | APOA5 |
| PSMA3 | NUP93 | CYP7A1 | GAL |
| FKBP1B | TNXA | DICER1 | TRH |
| ANKRD26 | DNMT1 | PAGR1 | DGUOK |
| TGIF1 | CENPB | FGF9 | BSG |
| DCTN1 | FOXL2 | CD1E | RHOC |
| PTHLH | SH2D1A | GAS5 | POC5 |
| CYP19A1 | TGFBR3 | DOCK8 | SLC6A3 |
| EMX2 | CD46 | PVALB | ALPK3 |
| DTNA | ATP7B | CHKA | CDK4 |
| NTRK2 | MECOM | SRRT | IGFBP1 |
| TRPV2 | MIR33A | MFN2 | ZEB1 |
| APLN | CLN8 | FOXO3 | TRAPPC11 |
| AHSG | CD22 | NT5E | SSB |
| MSN | CFAP47 | NAGS | CRHR1 |
| PLCZ1 | SRF | SPTAN1 | CTRC |
| OBSCN | CXCR5 | NRAP | ADCY9 |
| PGF | TLR8 | CEP85L | ATP6V1G2-DDX39B |
| TAGLN | WNT5A | COL6A1 | TREM1 |
| PPP1R12A | LUC7L2 | NID1 | CYSLTR2 |
| MMP14 | FOXG1 | ISG15 | SYNJ1 |
| HAVCR2 | ACAN | MGAM | HOPX |
| MSTN | CLCN1 | AQP4 | PTPA |
| TNNC2 | GHRH | COL9A1 | ID1 |
| CALR3 | MSLN | IDO1 | UBE2N |
| ROCK1 | RPS17 | OMP | ADSS1 |
| AGRN | CFLAR | PYGM | COL18A1 |
| MAGI2 | IL15RA | MALT1 | PDPN |
| RNU4ATAC | PCNA | MYOM1 | DRD2 |
| MIR10A | TNFSF12 | RHCE | NGF |
| CDKN2B | COG2 | SLC25A13 |  |

**Supplementary Table 3**: A total of 167 potential targets of 163 candidate compounds in WBT formula were obtained by network pharmacology.

| MOL ID | Molecule Name | Number | Gene names |
| --- | --- | --- | --- |
| MOL000006 | Luteolin | 37 | AKT1, APP, AR, BCL2L1, CASP3, CASP7, CASP9, CD40LG, CDKN1A, EGFR, ERBB2, GSTP1, HMOX1, IFNG, IL2, IL4, IL6, INSR, JUN, MAPK1, MDM2, MET, MMP1, MMP2, MMP9, NFKBIA, PCNA, PPARG, PRSS1, PTGS1, PTGS2, RELA, TNFSF15, TOP1, TP63, TYR, VEGFA |
| MOL000033 | (3S,8S,9S,10R,13R,14S,17R)-10,13-dimethyl-17-[(2R,5S)-5- propan-2-yloctan-2-yl]-2,3,4,7,8,9,11,12,14,15,16,17-dodecahydro-1H-cyclopenta[a]phenanthren-3-ol | 1 | PGR |
| MOL000073 | Ent-Epicatechin | 2 | PTGS1, PTGS2 |
| MOL000098 | Quercetin | 84 | ACHE, ADRB2, AKT1, ALOX5, AR, BAX, BCL2, BCL2L1, CASP3, CASP8, CASP9, CAV1, CCND1, CD40LG, CDKN1A, CHUK, COL1A1, COL3A1, CRP, CTSD, CXCL10, CXCL2, CYP1A1, CYP3A4, E2F1, EGF, EGFR, ERBB2, F3, FOS, GABRA1, GJA1, GSTM1, GSTP1, HIF1A, HMOX1, HSPB1, ICAM1, IFNG, IGF2, IGFBP3, IL10, IL1A, IL2, IL6, INSR, IRF1, JUN, KCNH2, MAPK1, MMP1, MMP2, MMP3, MMP9, MPO, MYC, NFE2L2, NFKBIA, NQO1, NR1I3, PARP1, PLAT, PLAU, PON1, PPARA, PPARG, PRSS1, PTGS1, PTGS2, RAF1, RELA, RUNX2, SCN5A, SELE, SERPINE1, SOD1, SPP1, STAT1, THBD, TNFSF15, TOP1, TP63, VCAM1, VEGFA |
| MOL000173 | Wogonin | 28 | ADRB2, AKT1, AR, BAX, BCL2, CASP3, CASP9, CCL2, CCND1, CDKN1A  , CXCL8, ESR1, FN1  , GABRA1, IL6, JUN, KDR, MAPK14, MMP1, PPARG, PRKCD, PRSS1, PTGS1, PTGS2, RELA, SCN5A, TNFSF15, TP63 |
| MOL000211 | Mairin | 1 | PGR |
| MOL000228 | (2R)-7-hydroxy-5-methoxy-2-phenylchroman-4-one | 9 | ADRB2, CHRM3, ESR1, GABRA1, PTGS1, PTGS2, SCN5A, SLC6A3, SLC6A4 |
| MOL000239 | Jaranol | 6 | AR, ESR2, PRSS1, PTGS1, PTGS2, SCN5A |
| MOL000296 | Hederagenin | 7 | ADH1C, CHRM3, GABRA1, PGR, PTGS1, PTGS2, SCN5A |
| MOL000354 | Isorhamnetin | 13 | ACHE, AR, CCNA2, ESR1, ESR2, GABRA1, MAPK14, NCF1, PPARG, PRSS1, PTGS1, PTGS2, RELA |
| MOL000358 | Beta-sitosterol | 16 | ADRB2, BAX, BCL2, CASP3, CASP8, CASP9, CHRM3, GABRA1, JUN, KCNH2, PGR, PON1, PTGS1, PTGS2, SCN5A, SLC6A4 |
| MOL000359 | Sitosterol | 2 | NR3C2, PGR |
| MOL000371 | 3,9-di-O-methylnissolin | 11 | ACHE, ADRB1, ADRB2, CHRM3, ESR1, GABRA1, HTR3A, PRSS1, PTGS1, PTGS2, SCN5A |
| MOL000378 | 7-O-methylisomucronulatol | 17 | ADRB1, ADRB2, AR, CCNA2, CHRM3, ESR1, ESR2, GABRA1, KCNH2, MAPK14, PPARG, PRSS1, PTGS1, PTGS2, SCN5A, SLC6A3, SLC6A4 |
| MOL000379 | 9,10-dimethoxypterocarpan-3-O-β-D-glucoside | 1 | PTGS2 |
| MOL000380 | (6aR,11aR)-9,10-dimethoxy- 6a,11a-dihydro-6H-benzofurano[3,2-c]chromen-3-ol | 10 | ACHE, ADRB2, CHRM3, ESR1, GABRA1, HTR3A, PRSS1, PTGS1, PTGS2, SCN5A |
| MOL000387 | Bifendate | 4 | KDR, MET, PTGS1, PTGS2 |
| MOL000392 | Formononetin | 11 | ADRB2, AR, ESR1, ESR2, IL4, JUN, PPARG, PTGS1, PTGS2, SLC6A3, SLC6A4 |
| MOL000417 | Calycosin | 10 | ADRB2, AR, CCNA2, ESR1, ESR2, MAPK14, PPARG, PRSS1, PTGS1, PTGS2 |
| MOL000422 | Kaempferol | 30 | ACHE, AKT1, ALOX5, AR, BAX, BCL2, CASP3, CYP1A1, CYP3A4, GABRA1, GSTM1, GSTP1, HMOX1, ICAM1, IKBKB, INSR, JUN, MAPK8, MMP1, NR1I3, PPARG, PRSS1, PTGS1, PTGS2, RELA, SELE, SLPI, STAT1,  TNFSF15, VCAM1 |
| MOL000442 | 1,7-Dihydroxy-3,9-dimethoxy pterocarpene | 2 | PRSS1, PTGS2 |
| MOL000449 | Stigmasterol | 12 | ADH1C, ADRB1, ADRB2, CHRM3, GABRA1, NR3C2, PGR, PLAU, PTGS1, PTGS2, SCN5A, SLC6A3 |
| MOL000492 | (+)-catechin | 4 | CAT, ESR1, PTGS1, PTGS2 |
| MOL000493 | Campesterol | 3 | PGR, PTGS1, PTGS2 |
| MOL000519 | Coniferin | 8 | ADRB2, AR, CCNA2, CHRM3, ESR1, PPARG, PTGS2, SCN5A |
| MOL000525 | Norwogonin | 4 | AR, PPARG, PTGS1, PTGS2 |
| MOL000552 | 5,2'-Dihydroxy-6,7,8-trimethoxyflavone | 8 | AR, ESR2, KCNH2, KDR, PRSS1, PTGS1 , PTGS2, SCN5A |
| MOL000569 | digallate | 1 | PTGS2 |
| MOL001323 | Sitosterol alpha1 | 5 | ADH1C, GABRA1, NR3C2, PGR, PTGS2 |
| MOL001328 | 2,3-didehydro GA70 | 4 | GABRA1, PRSS1, PTGS1, PTGS2 |
| MOL001329 | 2,3-didehydro GA77 | 2 | GABRA1, PTGS2 |
| MOL001340 | GA120 | 3 | CHRM3, GABRA1, PTGS2 |
| MOL001342 | GA121-isolactone | 1 | PGR |
| MOL001344 | GA122-isolactone | 1 | PGR |
| MOL001349 | 4a-formyl-7alpha-hydroxy-1-methyl-8-methylidene-4aalpha,4bbeta-gibbane-1alpha,10beta-dicar-boxylic acid | 2 | NR3C2, PGR |
| MOL001351 | Gibberellin A44 | 2 | GABRA1, NR3C2 |
| MOL001352 | GA54 | 1 | PTGS2 |
| MOL001353 | GA60 | 1 | GABRA1 |
| MOL001355 | MOL001355 | 2 | GABRA1, PTGS2 |
| MOL001358 | Gibberellin 7 | 5 | ADRB2, CHRM3, PTGS2, SLC6A3, SLC6A4 |
| MOL001360 | GA77 | 1 | GABRA1 |
| MOL001361 | GA87 | 1 | PTGS2 |
| MOL001368 | 3-O-p-coumaroylquinic acid | 2 | PTGS1, PTGS2 |
| MOL001458 | Coptisine | 7 | AR, ESR1, KCNH2, PRSS1, PTGS1, PTGS2, SCN5A |
| MOL001490 | Bis[(2S)-2-ethylhexyl] benzene-1,2-dicarboxylate | 1 | SCN5A |
| MOL001494 | Mandenol | 2 | PTGS1, PTGS2 |
| MOL001601 | 1,2,5,6-tetrahydrotanshinone | 9 | ADRB2, CHRM3, GABRA1, HTR3A, PTGS1, PTGS2, SCN5A, SLC6A3, SLC6A4 |
| MOL001659 | Poriferasterol | 2 | NR3C2, PGR |
| MOL001689 | Acacetin | 13 | ADRB2, AR, BAX, BCL2, CASP3, CASP8, CDKN1A, FASLG, PRSS1, PTGS1, PTGS2, RELA, TP63 |
| MOL001755 | 24-Ethylcholest-4-en-3-one | 2 | NR3C2, PGR |
| MOL001771 | Poriferast-5-en-3beta-ol | 1 | PGR |
| MOL001942 | Isoimperatorin | 1 | PTGS2 |
| MOL002135 | Myricanone | 12 | ADRB2, AR, CCNA2, ESR1, ESR2, KCNH2, KDR, MAPK14, PPARG, PTGS1, PTGS2, SCN5A |
| MOL002140 | Perlolyrine | 1 | PTGS2 |
| MOL002157 | Wallichilide | 3 | NR3C1, NR3C2, PTGS2 |
| MOL002222 | Sugiol | 6 | ACHE, ADRB2, CHRM3, DRD2, PTGS2, SCN5A |
| MOL002268 | Rhein | 3 | JUN, PTGS1, PTGS2 |
| MOL002563 | Galangin | 7 | AR, BCL2, CYP1A1, GSTP1, PPARG, PTGS1, PTGS2 |
| MOL002651 | Dehydrotanshinone II A | 9 | ACHE, ADRB2, AR, CHRM3, ESR1, GABRA1, PPARG, PTGS2, SCN5A |
| MOL002670 | Cavidine | 9 | ADRB1, ADRB2, CHRM3, HTR3A, KCNH2, PTGS1, PTGS2, SCN5A, SLC6A4 |
| MOL002714 | Baicalein | 17 | AKT1, AR, BAX, BCL2, CASP3, CYCS, FOS, HIF1A, IGF2, MMP9, MPO, PRSS1, PTGS1, PTGS2, RELA, TP63, VEGFA |
| MOL002879 | Diop | 3 | ADRB2, CHRM3, SCN5A |
| MOL002897 | Epiberberine | 5 | AR, ESR1, KCNH2, PRSS1, PTGS2 |
| MOL002909 | 5,7,2,5-tetrahydro-xy-8,6-dimethoxyflavone | 3 | AR, PRSS1, PTGS2 |
| MOL002910 | Carthamidin | 2 | PTGS1, PTGS2 |
| MOL002913 | Dihydrobaicalin_qt | 2 | PTGS1, PTGS2 |
| MOL002914 | Eriodyctiol (flavanone) | 2 | PTGS1, PTGS2 |
| MOL002915 | Salvigenin | 6 | ACHE, ADRB2, PRSS1, PTGS1, PTGS2, SCN5A |
| MOL002917 | 5,2',6'-Trihydroxy-7,8-dimethoxy-flavone | 6 | AR, ESR2, PRSS1, PTGS1, PTGS2, SCN5A |
| MOL002925 | 5,7,2',6'-Tetrahydr -oxyflavone | 3 | AR, PTGS1, PTGS2 |
| MOL002927 | Skullcapflavone II | 8 | AR, CACNA2D1, KCNH2, KDR, PRSS1, PTGS1, PTGS2, SCN5A |
| MOL002928 | Oroxylin a | 10 | ADRB2, AR, BCL2, CASP3, CYP2C9, IL6, PRSS1, PTGS1, PTGS2, SCN5A |
| MOL002932 | Panicolin | 6 | AR, ESR2, PRSS1, PTGS1, PTGS2, SCN5A |
| MOL002933 | 5,7,4'-Trihydroxy-8-methoxyflavone | 7 | AR, ESR1, MAPK14, PPARG, PRSS1, PTGS1, PTGS2 |
| MOL002934 | NEOBAICALEIN | 8 | AR, ESR1, ESR2, KCNH2, PPARG, PRSS1, PTGS2, SCN5A |
| MOL002937 | DIHYDROOR OXYLIN | 4 | ADRB2, PTGS1, PTGS2, SCN5A |
| MOL003036 | ZINC03978781 | 2 | NR3C2, PGR |
| MOL003578 | Cycloartenol | 1 | NR3C2 |
| MOL004172 | (1R)-1-[(2S,4aR,4bS,7R,8aS)-7-hydroxy-2,4b,8,8-tetramethyl-4,4a,5,6,7,8a,9,10-octahydro-3H-phenanthren-2-yl]ethane-1,2-diol | 1 | PGR |
| MOL004177 | 15alpha-Hydroxy-ent-kaur-16-en-19-oic acid | 2 | NR3C2, PGR |
| MOL004179 | Vernolic acid | 1 | PTGS2 |
| MOL004180 | Coronaridine | 8 | ADRB2, CHRM3, DRD2, GABRA1, HTR3A, PTGS1, SCN5A, SLC6A4 |
| MOL004184 | Siegesesteric acid II | 2 | NR3C2, PGR |
| MOL004185 | Siegesmethyletheric acid | 2 | NR3C2, PGR |
| MOL004355 | Spinasterol | 2 | NR3C2, PGR |
| MOL005030 | Gondoic acid | 1 | PTGS1 |
| MOL005100 | 5,7-dihydroxy-2-(3-hydroxy-4-methoxyphenyl)chroman-4-one | 3 | PTGS1, PTGS2, SCN5A |
| MOL005603 | Heptyl phthalate | 1 | ADRB2 |
| MOL006936 | 10,13-eicosadienoic | 1 | PTGS1 |
| MOL006957 | (3S,6S)-3-(benzyl)-6-(4-hydroxybenzyl)piperazine-2,5-quinone | 3 | ADRB2, AR, PTGS2 |
| MOL006967 | Beta-D-Ribofuranoside, xanthine-9 | 1 | PTGS2 |
| MOL007036 | 5,6-dihydroxy-7-isopropyl-1,1-dimethyl-2,3- dihydrophenanthren-4-one | 6 | ACHE, ADRB2, CHRM3, PTGS1, PTGS2, SCN5A |
| MOL007041 | 2-isopropyl-8-methylphenan- threne-3,4-dione | 13 | ADRB2, AR, CCNA2, CHRM3, ESR1, GABRA1, HTR3A, PPARG, PTGS1, PTGS2, SCN5A, SLC6A3, SLC6A4 |
| MOL007045 | 3α-hydroxytanshinoneⅡa | 5 | ACHE, ADRB2, PRSS1, PTGS2, SCN5A |
| MOL007048 | (E)-3-[2-(3,4-dihydroxyphenyl)-7-hydroxy-benzofuran-4-yl]acrylic acid | 1 | PTGS2 |
| MOL007049 | 4-methylenemiltirone | 12 | ADRB2, AR, CHRM3, DRD2, ESR1, GABRA1, PPARG, PTGS1, PTGS2, SCN5A, SLC6A3, SLC6A4 |
| MOL007050 | 2-(4-hydroxy-3-methoxyphenyl)-5-(3-hydroxypropyl)-7-methoxy-3-benzofurancarboxaldehyde | 6 | AR, CCNA2, ESR1, ESR2, MAPK14, PPARG |
| MOL007058 | Formyltanshinone | 2 | AR, PTGS2 |
| MOL007059 | 3-beta-Hydroxymethyllenetanshiquinone | 4 | ACHE, ADRB2, PRSS1, PTGS2 |
| MOL007061 | Methylenetanshinquinone | 8 | ACHE, ADRB2, CHRM3, GABRA1, PRSS1, PTGS2, SCN5A, SLC6A4 |
| MOL007063 | Przewalskin a | 2 | NR3C1, NR3C2 |
| MOL007064 | Przewalskin b | 4 | NR3C1, NR3C2, PGR, PTGS2 |
| MOL007068 | Przewaquinone B | 2 | PRSS1, PTGS2 |
| MOL007069 | Przewaquinone c | 7 | ACHE, ADRB2, CHRM3, GABRA1, PTGS1, PTGS2, SCN5A |
| MOL007070 | (6S,7R)-6,7-dihydroxy-1,6-dimethyl-8,9-dihydro-7H-naphtho[8,7-g]benzofuran-10,11-dione | 3 | ACHE, PRSS1, PTGS2 |
| MOL007071 | Przewaquinone f | 2 | PRSS1, PTGS2 |
| MOL007077 | Sclareol | 1 | PTGS2 |
| MOL007079 | Tanshinaldehyde | 4 | ACHE, ADRB2, PRSS1, PTGS2 |
| MOL007081 | Danshenol B | 3 | NR3C1, PGR, PTGS2 |
| MOL007082 | Danshenol A | 4 | KCNH2, PTGS1, PTGS2, SCN5A |
| MOL007085 | Salvilenone | 6 | AR, ESR1, ESR2, HTR3A, PTGS1, PTGS2 |
| MOL007088 | Cryptotanshinone | 14 | ADRB2, APP, BCL2L1, CCND1, CHRM3, EDN3, GABRA1, PGR,  PTGS1, PTGS2, RELA, SCN5A, STAT3, TNFSF15 |
| MOL007093 | Dan-shexinkum d | 12 | ACHE, ADRB2, AR, CCNA2, ESR1, ESR2, KCNH2, PPARG, PRSS1, PTGS1, PTGS2, SCN5A |
| MOL007094 | Danshenspiroketallactone | 9 | ACHE, ADRB2, CHRM3, ESR1, GABRA1, PTGS1, PTGS2, SCN5A, SLC6A4 |
| MOL007098 | Deoxyneocryptotanshinone | 7 | ADRB2, AR, CHRM3, ESR1, PTGS1, PTGS2, SCN5A |
| MOL007100 | Dihydrotanshinlactone | 15 | ACHE, ADRB2, AR, CCNA2, CHRM3, ESR1, GABRA1, HTR3A, PPARG, PRSS1, PTGS1, PTGS2, SCN5A, SLC6A3, SLC6A4 |
| MOL007105 | Epidanshenspiroketallactone | 8 | ADRB2, CHRM3, ESR1, GABRA1, PTGS1, PTGS2, SCN5A, SLC6A4 |
| MOL007107 | C09092 | 4 | ACHE, ADRB2, CHRM3, SCN5A |
| MOL007108 | Isocryptotanshi-none | 11 | ACHE, ADRB2, AR, CHRM3, DRD2, ESR1, GABRA1, PRSS1, PTGS1, PTGS2, SCN5A |
| MOL007111 | Isotanshinone II | 10 | ACHE, ADRB2, AR, CCNA2, CHRM3, ESR1, ESR2, GABRA1, PTGS2, SCN5A |
| MOL007121 | Miltipolone | 2 | ACHE, ESR1 |
| MOL007122 | Miltirone | 8 | ADRB2, AR, CHRM3, ESR1, PTGS1, PTGS2, SCN5A, SLC6A3 |
| MOL007124 | Neocryptotanshinone ii | 11 | ADRB2, AR, CCNA2, CHRM3, ESR1, GABRA1, PTGS1, PTGS2, SCN5A, SLC6A3, SLC6A4 |
| MOL007125 | Neocryptotanshinone | 6 | ADRB2, CHRM3, PPARG, PTGS1, PTGS2, SCN5A |
| MOL007127 | 1-methyl-8,9-dihydro-7H-naphtho[5,6-g]benzofuran-6,10,11-trione | 7 | ACHE, ADRB2, CHRM3, GABRA1, PTGS1, PTGS2, SCN5A |
| MOL007130 | Prolithospermic acid | 5 | AR, ESR1, PRSS1, PTGS1, PTGS2 |
| MOL007132 | (2R)-3-(3,4-dihydroxyphenyl)-2-[(Z)-3-(3,4-dihydroxyphenyl)acryloyl]oxy-propionic acid | 6 | AR, CCNA2, ESR1, PPARG, PRSS1, PTGS2 |
| MOL007141 | Salvianolic acid g | 1 | PTGS2 |
| MOL007142 | Salvianolic acid j | 1 | PRSS1 |
| MOL007145 | Salviolone | 5 | CHRM3, HTR3A, PTGS1, PTGS2, SCN5A |
| MOL007150 | (6S)-6-hydroxy-1-methyl-6-methylol-8,9-dihydro-7H-naphtho[8,7-g]benzofuran-10,11-quinone | 3 | ACHE, PRSS1, PTGS2 |
| MOL007151 | Tanshindiol B | 2 | ACHE, PTGS2 |
| MOL007152 | Przewaquinone E | 2 | ACHE, PTGS2 |
| MOL007154 | Tanshinone iia | 21 | ACHE, ADRB2, BCL2, CASP3, CDKN1A, CHRM3, CYP1A1, CYP3A4, EDN3, EDNRA, FOS, ITGB3, JUN, MMP9, MYC, NFKBIA, NPM1, PTGS2, RELA, SCN5A, TP63 |
| MOL007155 | (6S)-6-(hydroxymethyl)-1,6-dimethyl-8,9-dihydro-7H-naphtho[8,7-g]benzofuran-10,11-dione | 5 | ACHE, ADRB2, PRSS1, PTGS2, SCN5A |
| MOL008206 | Moslosooflavone | 10 | ADRB2, AR, ESR2, GABRA1, MAPK14, PPARG, PRSS1, PTGS1, PTGS2, SCN5A |
| MOL010003 | 7beta-angeloyloxyoplopa-3(14)Z,8(10)-dien-2-one | 1 | PTGS2 |
| MOL010004 | 7beta-(4-methylsenecioyloxy)oplopa-3(14)E,8(10)-dien-2-one | 1 | PTGS2 |
| MOL010006 | 7beta-senecioyloxyoplopa-3(14)Z,8(10)-dien-2-one | 2 | NR3C1, NR3C2 |
| MOL010007 | la-7beta-di(4-methulsenecioyloxy) oplopa-3(14)Z,8(10)-dien  -2-one | 1 | NR3C1 |
| MOL010013 | 7beta-(3-ethyl-cis-crotonoyloxy)-14-hydroxy-notonipetranone | 1 | PTGS2 |
| MOL010014 | 14-acetoxy-7beta-angeloyloxy-notonipetranone | 2 | NR3C2, PTGS2 |
| MOL010015 | 14-acetoxy-7beta-senecioyloxy-notonipetranone | 1 | NR3C2 |
| MOL010023 | Senkirkine | 1 | PTGS2 |
| MOL010028 | Tussilagin | 2 | NR3C2, PTGS2 |
| MOL010055 | Femara | 2 | PTGS1, PTGS2 |
| MOL010058 | Methyl 3-o-caffeoylquinate | 1 | PTGS2 |
| MOL010428 | 16beta,17-dihydroxy-(-)-kauran-19-ate-beta-D-glucose ester_qt | 1 | NR3C2 |
| MOL010470 | Rabdosinatol | 1 | NR3C2 |
| MOL010473 | Shionone | 1 | PGR |
| MOL012245 | 5,7,4'-trihydroxy-6-methoxyflavanone | 2 | PTGS1, PTGS2 |
| MOL012246 | 5,7,4'-trihydroxy-8-methoxyflavanone | 2 | PTGS1, PTGS2 |
| MOL012266 | Rivularin | 8 | AR, ESR2, KCNH2, KDR, PRSS1, PTGS1, PTGS2, SCN5A |
| MOL013281 | 6,8-Dihydroxy-7-methoxyxanthone | 4 | ADRB2, MAPK14, PTGS1, PTGS2 |
| MOL013287 | Physovenine | 14 | ACHE, ADRB2, AR, CCNA2, CHRM3, ESR1, ESR2, GABRA1, PRSS1, PTGS1, PTGS2, SCN5A, SLC6A3, SLC6A4 |
| MOL013288 | Picralinal | 2 | AR, SCN5A |
| - | Xanthine | 9 | AR, CASP3, CASP7, CTSD, F2RL1, JUN, PLA2G1B, PTGS2, STAT3 |
| - | Xanthinin | 47 | ACHE, ADORA2A, ALPL, AR, BCL2L1, CCND1, CDK6, CSF1R, CTSB, CTSL, CXCR3, CYP11B2, CYP19A1, DPP4, F10, F13A1, FLT1, HTR2B, IDH1, IKBKB, IL1B, JAK1, JAK2, JAK3, KCNJ5, KDM4C, MALT1, MAPK14, MAPK8, MME, MMP1, MMP2, MMP3, MMP9, NOS1, P2RX7, PARP1, PDE4D, PDGFRB, PPARA, PRKD1, PTGS2, SIRT1, TGM2, TLR9, TTR |
| - | 4-guanidino-1-butanol | 3 | ADRB2, GBA, PLG |
| - | Cholesterol | 12 | ACHE, AR, CCR1, CYP19A1, ESR1, ESR2, G6PD, HMGCR, PRKCD,  SERPINA6, SHBG, SLC6A4 |
| - | Cholesteryl ferulate | 4 | EDNRA, ELANE, HDAC2, MET |
| - | Guanine(1,7-dihydro-form) | 1 | ACHE |
| - | Hyrcanoside | 9 | AR, CASP3, CASP7, CTSD, F2RL1, JUN, PLA2G1B, PTGS2, STAT3 |
